# Evaluation of reporting quality of cohort studies using real-world data based on RECORD: systematic review

**DOI:** 10.1186/s12874-023-01960-2

**Published:** 2023-06-29

**Authors:** Ran Zhao, Wen Zhang, ZeDan Zhang, Chang He, Rong Xu, XuDong Tang, Bin Wang

**Affiliations:** 1 Institute of Information on Traditional Chinese Medicine, China Academy of Chinese Medical Sciences, Beijing, China; 2 Institute of Basic Research in Clinical Medicine, China Academy of Chinese Medical Sciences, Beijing, China; 3 Traditional Chinese Medicine Data Center, China Academy of Chinese Medical Sciences, Beijing, China; 4 Guang’anmeng Hospital, China Academy of Chinese Medical Sciences, Beijing, China; 5 China Academy of Chinese Medical Sciences, Beijing, China

**Keywords:** Real world data, Real world evidence, Cohort studies, Reporting quality, The RECORD statement

## Abstract

**Objective:**

Real-world data (RWD) and real-world evidence (RWE) have been paid more and more attention in recent years. We aimed to evaluate the reporting quality of cohort studies using real-world data (RWD) published between 2013 and 2021 and analyze the possible factors.

**Methods:**

We conducted a comprehensive search in Medline and Embase through the OVID interface for cohort studies published from 2013 to 2021 on April 29, 2022. Studies aimed at comparing the effectiveness or safety of exposure factors in the real-world setting were included. The evaluation was based on the REporting of studies Conducted using Observational Routinely-collected health Data (RECORD) statement. Agreement for inclusion and evaluation was calculated using Cohen’s kappa. Pearson chi-square test or Fisher’s exact test and Mann-Whitney U test were used to analyze the possible factors, including the release of RECORD, journal IFs, and article citations. Bonferroni’s correction was conducted for multiple comparisons. Interrupted time series analysis was performed to display the changes in report quality over time.

**Results:**

187 articles were finally included. The mean ± SD of the percentage of adequately reported items in the 187 articles was 44.7 ± 14.3 with a range of 11.1–87%. Of 23 items, the adequate reporting rate of 10 items reached 50%, and the reporting rate of some vital items was inadequate. After Bonferroni’s correction, the reporting of only one item significantly improved after the release of RECORD and there was no significant improvement in the overall report quality. For interrupted time series analysis, there were no significant changes in the slope (p = 0.42) and level (p = 0.12) of adequate reporting rate. The journal IFs and citations were respectively related to 2 areas and the former significantly higher in high-reporting quality articles.

**Conclusion:**

The endorsement of the RECORD cheklist was generally inadequate in cohort studies using RWD and has not improved in recent years. We encourage researchers to endorse relevant guidelines when utilizing RWD for research.

**Supplementary Information:**

The online version contains supplementary material available at 10.1186/s12874-023-01960-2.

## Introduction

Real-world data (RWD) is defined as the data relating to patient health status and/or the delivery of health care that is routinely collected from a variety of sources, such as patient registries, electronic medical records (EMRs), electronic health records (EHRs), insurance claims, and patient health records [1, 2], by the US Food and Drug Administration [3]. Real-world evidence (RWE) generated by studies using RWD could be the complement of clinical trials for observing the effectiveness or safety of drugs, products, operations, or any other treatment measures and play an increasingly significant role in decision-making while it is more reflective of clinical practice compared to the evidence generated by randomized trials [4–7]. However, the reporting of studies using RWD currently exists with the problems of inadequate and lack of transparency, which will limit the reproducibility and replicability of studies and arouse concerns, doubts, and reductions of confidence in RWE [8–11]. Some reporting guidelines have been established to standardize studies reports and promote the quality of RWE [9, 12–15].

The Reporting of studies Conducted using Observational Routinely-collected health Data (RECORD) statement [12], which is an extension of the Strengthening the Reporting of Observational studies in Epidemiology (STROBE) statement [16], was created to address the specific reporting issues of studies using routinely-collected data. Previous studies have shown the reporting of observational studies was usually insufficient whether based on RECORD or STROBE [17–19].

According to the RECORD checklist, we evaluated the quality of reporting on specific aspects of studies using RWD, such as codes and algorithms, data linkage and cleaning, and discussion of peculiar limitations. We concentrated on cohort studies that compared the effectiveness or safety of exposure factors, since cohort studies are helpful to provide evidence indicating causality, the strength of correlation between exposure factors and outcomes, and can usually produce highly generalizable results [20, 21] while comparative research could complement or assess the evidence originated from randomized trials and inform decisions about health policy and clinical care [22]. The RECORD checklist was transformed into a series of questions for convenient and accurate evaluation.

We aimed to evaluate the reporting quality of cohort studies using RWD published from 2013 to 2021 and analyze the possible factors of reporting quality. Comparative analyses were conducted to ascertain whether the report quality is related to the release of the RECORD statement, journal impact factors (IFs), and citations of individual articles.

**Fig. 1:**
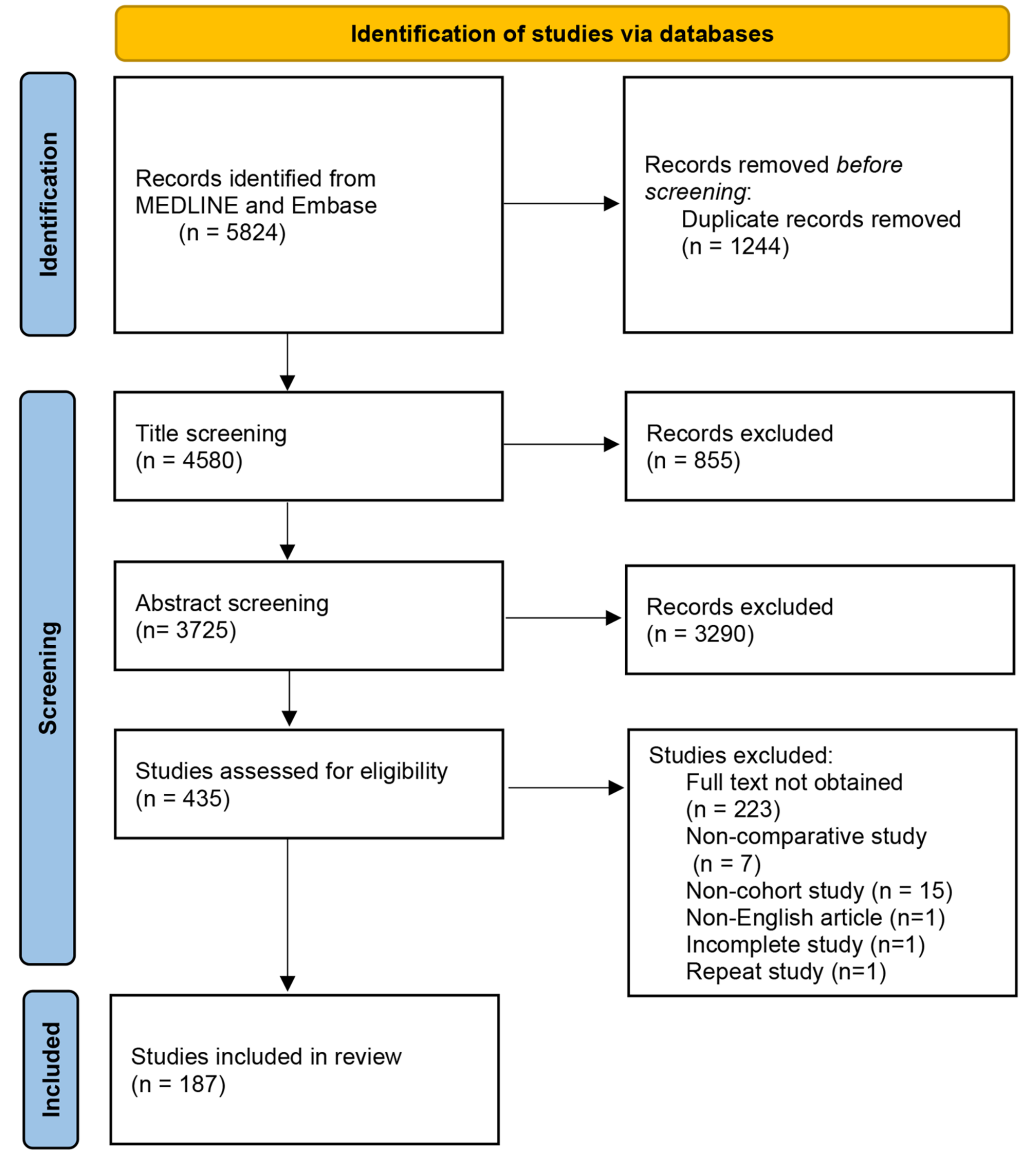
Flow diagram

## Methods

### Eligibility of studies

We selected cohort studies that aimed to compare the effectiveness and safety of the exposure factors and used real-world data, including prospective, retrospective, and both. For example, we excluded studies that classified populations by disease type [23]. Studies on morbidity, mortality, or hazard factors of diseases and economic benefits or medical expenses of exposure factors were excluded. Articles whose data were not clearly derived from the real world, or not for the purpose of obtaining real-world evidence were also excluded. We only considered the English language articles published between 2013 and 2021. For the wider applicability of research results, no more restrictions on research diseases or participants, exposure measures, and the settings of control groups.

**Table 1:** Characteristics of included studies

Characteristics	Studies [n (%)]	Characteristics	Studies [n (%)]
Country		Publication Year	
USA	56(29.9)	2013	8(4.3)
Germany	16(8.6)	2014	10(5.3)
Japan	14(7.5)	2015	15(8)
Korea	13(7)	2016	14(7.5)
China	11(5.9)	2017	24(12.8)
Denmark	10(5.3)	2018	26(13.9)
Spain	9(4.8)	2019	29(15.5)
Italy	9(4.8)	2020	31(16.6)
Other	49(26.2)	2021	30(16)
**Diseases**		**Type of Therapies**	
Cancer	32(16.7)	Drug	106(56.7)
Diabetes Mellitus	22(11.5)	Antibody	26(13.9)
Arthritis	15(7.8)	Device	22(11.8)
Coronary Disease	15(7.8)	Surgery	16(8.6)
Chronic Hepatitis C	8(4.2)	Chemotherapy/Radiotherapy	6(3.2)
Myocardial Infarction	8(4.2)	Biologics	5(2.7)
Atrial Fibrillation	7(3.6)	Allograft	3(1.6)
Neoplasm	7(3.6)	Vaccination	2(1.1)
Multiple Sclerosis	6(3.1)	Acupuncture	1(0.5)
Hypertension	5(2.6)		
Psoriasis	5(2.6)		
Stroke	3(1.6)		
Diabetic Foot Ulcers	2(1)		
Heart Failure	2(1)		
Other	55(28.6)		
**Type of Data Sources**		**Number of databases**	
Multicenter Registry	116(62)	1	121(64.7)
Single center registry	6(3.2)	2	32(17.1)
EMR/EHR	34(18.2)	3	13(7)
Claims	11(5.9)	4	5(2.7)
Other	29(15.5)	≥ 5	16(8.6)
**Journal IF (n = 176)**		**Citations (n = 187)**	
Median (IQR)	3.54(2.4,5.55)	Median (IQR)	12(6,25)
Range	0.52–27.97	Range	0-548

IQR, interquartile range; EMR, electronic medical record; EHR, electronic health record; IF, impact factor.

Country means the country of the corresponding author; therapies involve exposure group and control group; journal IF is in the publication year of the article.

### Search strategy

We conducted a comprehensive search in Medline and Embase through the OVID interface for English language articles published between 2013 to 2021(Search on April 29, 2022). The indexing terms included study design, data sources of studies (e.g., “routinely collected data”, “health information system”, “electronic medical record”, “registry”), excluded publication types or article types (e.g., “review”, “protocol”, “meta-analysis”) and excluded outcomes. The search filters developed by Scottish Intercollegiate Guidelines Network [24] and the strategy developed by Lars G. Hemkens et al. [19] were integrated into our search strategy (available in S1 File).

**Table 2:** Reporting of cohort studies using RWD

Items	Brief Descriptions	Reporting [n (%)]			Not Applicable
		Yes	Partly Yes	No	
Title and abstract					
R1.1a	Type of data	182(97.3)		5(2.7)	
R1.1b	Name of databases	134(71.7)	2(1.1)	51(27.2)	
R1.2a	Geographic region	117(62.6)		70(37.4)	
R1.2b	Time frame	97(51.9)		90(48.1)	
R1.3	Linkage between databases	33(50)		33(50)	121
**Methods**					
R6.1	Population selection methods	75(40.1)	112(59.9)		
R6.2	Validation of codes/algorithms for population selection	21(28)		54(72)	112
R6.3	Graphical display of data linkage process	12(18.2)		54(81.8)	121
R7.1a	Codes/algorithms of exposures	28(15)	2(1)	157(84)	
R7.1b	Codes/algorithms of outcomes	43(23)	34(18.2)	11(5.9)	
R7.1c	Codes/algorithms of confounders and effect modifiers	23(12.3)	7(3.7)	157(84)	
R12.1	The extent of database accessed	153(81.8)		34(18.2)	
R12.2	Data-cleaning methods	65(34.8)		122(65.2)	
R12.3	Detail of data linkage	28(42.4)	28(42.4)	10(15.2)	121
**Results**					
R13.1	Detail of population selection	107(57.2)	19(10.2)	61(32.6)	
**Discussion**					
R19.1a	Inherent limitations	155(82.9)		32(17.1)	
R19.1b	Risk of misclassification	58(31)		129(69)	
R19.1c	Limitations of codes/algorithms and the validation	19(25.3)		56(74.7)	112
R19.1d	Implications of the missing variables and missing data	118(63.1)		69(36.9)	
R19.1e	Eligibility of results over time	34(18.2)		153(81.8)	
**Other information**					
R22.1a	Study protocol availability	44(23.5)		143(76.5)	
R22.1b	Raw data availability	38(20.3)		149(79.7)	
R22.1c	Supplementary materials availability	100(53.5)		87(46.5)	

RWD, real-world data

### Screening and data extraction

We imported search results into Note Express (Beijing Aegean Hailezhi Technology Co., Ltd., Beijing, China) for management, removing duplicated articles and screening. First, articles that did not conform to the eligibility criteria were excluded by independently screening the titles and abstracts by two reviewers (R.Z. and W.Z.). Subsequently, we downloaded all the available full texts of preliminary included articles. Two reviewers (R.Z. and W.Z.) read each full-text article to screen out the articles that were ultimately included and record the reason for exclusion. Cohen’s kappa was calculated to assess the agreement of manuscript level inclusion. Any discrepancies in the screening process were resolved via discussion or determined by a third author (one of B.W., Z.D.Z, C.H.).

We extracted the characteristics of each qualified article including the year of publication, country of the corresponding author, type of disease, journal name, journal IFs in the year of publication, citations, type of therapy, and type of data source. To guarantee the accuracy of the journal names and journal impact factors, we identified the journals by DOI, PMID, or site link provided in the articles and searched the ISSN of journals on the Web of Science to obtain the journal impact factors in the year of publication. Article citations were accessed on Google Scholar. Two reviewers (two of R.Z., W.Z., Z.D.Z, and C.H.) completed the process of extraction and recorded data in Microsoft Excel (Microsoft Corporation, USA). Any discrepancies were resolved via consensus.

**Table 3:** Reporting before and after the release of RECORD

Items	Reporting	Studies(n)		OR (95%CI)	*p-value*
		Pre-RECORD	Post-RECORD		
R1.1a	Yes Partly Yes/No	32 1	115 1	3.59(0.22,59.06)	0.395
R1.1b	Yes Partly Yes/No	26 7	84 32	0.71(0.28,1.79)	0.462
R1.2a	Yes Partly Yes/No	18 15	77 39	1.65(0.75,3.61)	0.212
R1.2b	Yes Partly Yes/No	16 17	64 52	1.31(0.6,2.84)	0.497
R1.3	Yes Partly Yes/No	6 10	26 16	2.71(0.83,8.89)	0.095
R6.1	Yes Partly Yes/No	14 19	51 65	1.07(0.49,2.33)	0.875
R6.2	Yes Partly Yes/No	6 8	12 39	0.41(0.12,1.42)	0.152
R6.3	Yes Partly Yes/No	3 13	9 33	1.18(0.28,5.07)	0.822
R7.1a	Yes Partly Yes/No	4 29	23 93	1.79(0.57,5.61)	0.443
R7.1b	Yes Partly Yes/No	8 25	30 86	1.09(0.44,2.68)	0.851
R7.1c	Yes Partly Yes/No	4 29	14 102	1(0.3,3.26)	1
R12.1	Yes Partly Yes/No	27 6	103 13	1.76(0.61,5.06)	0.289
R12.2	Yes Partly Yes/No	11 22	42 74	1.14(0.5,2.57)	0.761
R12.3	Yes Partly Yes/No	5 11	22 20	2.42(0.72,8.18)	0.149
R13.1	Yes Partly Yes/No	19 14	71 45	1.16(0.53,2.55)	0.71
R19.1a	Yes Partly Yes/No	27 6	96 20	1.07(0.39,2.92)	0.9
R19.1b	Yes Partly Yes/No	11 22	33 83	0.8(0.35,1.82)	0.587
R19.1c	Yes Partly Yes/No	6 8	12 39	0.41(0.12,1.42)	0.152
R19.1d	Yes Partly Yes/No	25 8	71 45	0.51(0.21,1.22)	0.123
R19.1e	Yes Partly Yes/No	8 25	21 95	0.69(0.27,1.74)	0.432
R22.1a	Yes Partly Yes/No	9 24	28 88	0.85(0.35,2.04)	0.713
R22.1b	Yes Partly Yes/No	0 33	36 80	--	**<0.001**
R22.1c	Yes Partly Yes/No	12 21	75 41	3.2(1.43,7.16)	**0.004** ^**a**^
Total		--	--	1.17(0.98,1.39)	0.082

RECORD, Reporting of studies Conducted using Observational Routinely-collected health Data.

Pre-RECORD refers to articles published in 2013–2015, Post-RECORD refers to articles published in 2018–2021.

^a^No significant difference after Bonferroni’s correction.

## Evaluation

Included articles were evaluated by the RECORD checklist, an extension of the STROBE checklist. Considering that some items of the RECORD checklist contain various aspects, we split and transformed the 13 items of the RECORD checklist into 23 questions that can be answered “yes”, “partly yes”, “no”, or “not applicable”. Reported as “yes” was considered adequate reporting while reported as “partly yes” or “no” was considered inadequate reporting. Some questions may not correspond to the RECORD checklist, but they are emphasized in the explanations of the RECORD statement and are necessary to be reported. For the split items, we renamed the different items with lowercase letters (e.g., R1.1 split to R1.1a, R1.1b). Five items appear to be inapplicable, in brief, three items (R1.3, R6.3, and R12.3) would be inapplicable if the study did not require database linkage and two items (R6.2 and R19.1e) would be inapplicable if the study did not report the codes or algorithms for population selection. We considered that other items should be reported in studies using RWD and therefore applied by default. The complete list of questions, descriptions, and examples can be obtained in S1 Table.

**Fig. 2:**
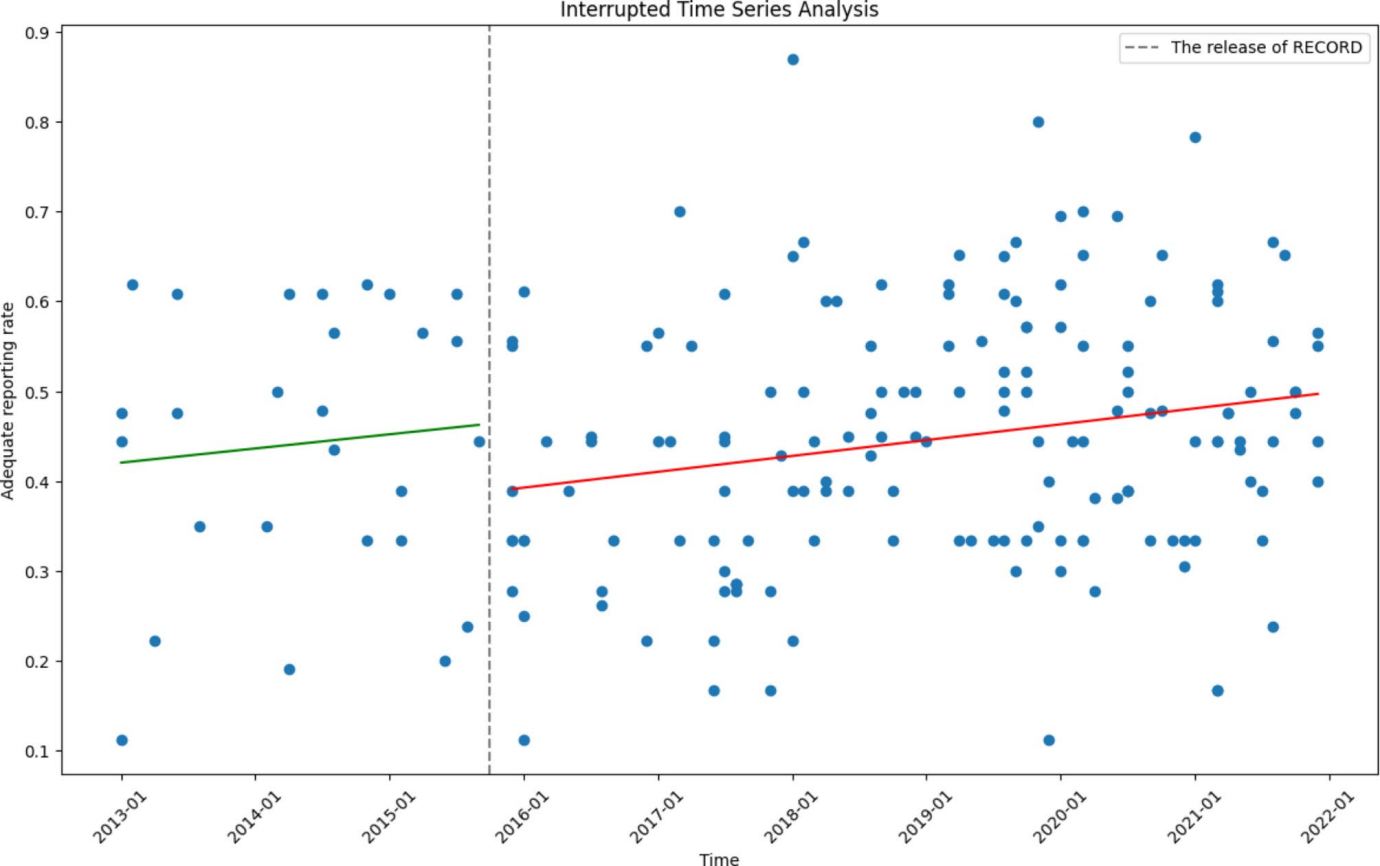
Trend of adequate reporting rate from 2013 to 2021

We randomly selected 10 included articles and four reviewers (R.Z., W.Z., Z.D.Z, C.H.) preliminary evaluated them through the constructed 23 questions. Afterwards every reviewer proposed problems and suggestions generated in the preliminary evaluation for a panel discussion to eliminate all discrepancies and ameliorate the 23 questions. Finally, two independent reviewers (R.Z. and W.Z.) evaluated each of included articles and integrated the results in Microsoft Excel (Microsoft Corporation, USA), any discrepancies were resolved via discussion or determined by two other authors (Z.D.Z, C.H.).Agreement for evaluation was als calculated using Cohen’s kappa.

**Table 4:** Correlations of journal IFs and citations with reporting quality

Items	Reporting	Journal IFs			Citations		
		Studies(n)	Median (IQR)	*p-value*	Studies(n)	Median (IQR)	*p-value*
R1.1a	Yes Partly Yes/No	171 5	3.58(2.39,5.55) 3.19(1.56,4.94)	0.49	182 5	12(6,26) 12(4.5,15)	0.458
R1.1b	Yes Partly Yes/No	125 51	3.71(2.46,5.52) 3.39(2.28,6.24)	0.841	134 53	12 (7,25.25) 12(4,25.5)	0.493
R1.2a	Yes Partly Yes/No	110 66	3.85(2.45,5.56) 3.3(2.35,5.54)	0.446	117 70	12(5.5,24.5) 12.5(7,26)	0.602
R1.2b	Yes Partly Yes/No	88 88	3.42(2.3,5.17) 3.79(2.46,6.11)	0.376	97 90	10(4.5,24.5) 15(7.75,26.25)	0.113
R1.3	Yes Partly Yes/No	33 32	4.4(2.65,5.36) 4.65(3.3,8.62)	0.322	33 33	10(4.5,30.5) 17(7,39.5)	0.16
R6.1	Yes Partly Yes/No	70 106	4.11(2.65,5.99) 3.24(2.26,5.22)	**0.04** ^**a**^	75 112	16(7,32) 10.5(5,19.75)	**0.035** ^**a**^
R6.2	Yes Partly Yes/No	20 50	4.01(3.14,6.13) 4.15(2.38,5.99)	0.326	21 55	23(9,60.5) 15.5(6.75,27.25)	0.088
R6.3	Yes Partly Yes/No	12 53	4.53(3.3,5.66) 4.4(2.9,6.73)	0.906	12 54	16.5(4.25,36.5) 13.5(5.75,35)	0.874
R7.1a	Yes Partly Yes/No	25 151	4.07(2.78,6.8) 3.4(2.32,5.48)	0.116	28 159	17(9.5,43.75) 11(6,23)	**0.02** ^**a**^
R7.1b	Yes Partly Yes/No	40 136	4.15(3.23,6.45) 3.24(2.23,5.49)	**0.014** ^**a**^	43 144	15(7,41) 11.5(5.25,23)	0.129
R7.1c	Yes Partly Yes/No	20 156	4.58(2.82,12.43) 3.42(2.35,5.42)	0.036	23 164	19(12,66) 10.5(5,23)	**0.001**
R12.1	Yes Partly Yes/No	145 31	3.94(2.55,5.71) 2.6(1.71,4.4)	**0.004** ^**a**^	153 34	12(6,27.5) 12.5(5.5,18.75)	0.534
R12.2	Yes Partly Yes/No	61 115	4.07(2.62,6.21) 3.28(2.32,5.1)	0.072	65 122	14(7,29.5) 11(5,23)	0.276
R12.3	Yes Partly Yes/No	28 37	5.16(3.61,8.46) 4.01(2.64,6.07)	0.086	28 38	15(4.25,51.5) 13(5,30)	0.559
R13.1	Yes Partly Yes/No	101 75	3.95(2.62,5.71) 3.19(2.14,5.49)	0.119	107 80	11(5,23) 15(6.25,26)	0.628
R19.1a	Yes Partly Yes/No	145 31	3.75(2.46,5.59) 3.15(1.95,5.49)	0.133	155 32	13(7,27) 8.5(3.25,15,75)	**0.01** ^**a**^
R19.1b	Yes Partly Yes/No	54 122	4.02(2.59,6.51) 3.47(2.34,5.22)	0.135	58 129	15(8,30) 10(5,23)	0.099
R19.1c	Yes Partly Yes/No	19 51	4.07(3.06,5.57) 4.15(2.45,6.29)	0.751	19 56	27(9,41) 15.5(6,27.75)	0.09
R19.1d	Yes Partly Yes/No	112 64	3.68(2.42,5.6) 3.39(2.33,5.42)	0.476	118 69	15(8,29.25) 9(3.5,18)	**0.001**
R19.1e	Yes Partly Yes/No	30 146	2.53(1.7,3.77) 3.98(2.55,5.66)	**0.001**	34 153	10.5(7.75,20.75) 12(5,25.5)	0.855
R22.1a	Yes Partly Yes/No	44 132	3.91(2.38,6.45) 3.47(2.41,5.42)	0.582	44 143	14.5(7,31.25) 11(6,25)	0.469
R22.1b	Yes Partly Yes/No	36 140	4.64(2.78,6.49) 3.26(2.33,5.19)	**0.022** ^**a**^	38 149	8(2,18) 14(7,26)	**0.015** ^**a**^
R22.1c	Yes Partly Yes/No	83 93	4.4(3.03,6.21) 2.65(1.85,4.43)	**<0.001**	87 100	12(6.25,25.75) 12(6,25)	0.681
Total	≥ 50% < 50%	65 111	4.2(2.97,6.02) 3.17(2.14,5.18)	**0.002**	72 115	16.5(8.25,36.5) 10(5,19)	**0.004** ^**a**^

IFs, impact factors; IQR, interquartile range

Journal IFs of 11 articles were unavailable and therefore not included in the analysis. Citations were obtained from Google Scholar on November 18.

^a^No significant difference after Bonferroni’s correction.

### Data analysis

We respectively performed horizontal and vertical calculations. For each item, the number of answers that were “yes”, “partly yes”, “no” and “not applicable” was calculated, as well as the percentage after removing the inapplicable items. For each article, the number of each type of answer was similarly calculated, as well as the rate of adequate reporting which was regarded as the overall quality of the individual articles.

To compare the reporting quality before and after the release of RECORD (released on October 6, 2015), we conducted a before-and-after analysis, with articles published from 2013 to 2015 defined as Pre-RECORD articles and articles published from 2018 to 2021 defined as Post-RECORD articles. Considering the time required to publish the article and the dissemination of RECORD, a two-year interval was allowed. In addition, we also conducted interrupted time series analysis (ITSA) to demonstrate the changes in adequate reporting rate over time and the differences before and after the release of RECORD.

The median and interquartile range (IQR) were used to describe continuous variables. We compared the reporting of each item of Pre-RECORD articles and Post-RECORD articles using the Pearson chi-square test or Fisher’s exact test and calculated odd ratios and 95% confidence intervals. Due to the non-normal distribution of the data, the Mann-Whitney U test was used to analyze the correlations of journal IFs and citations with reporting quality. We also set a 50% reporting rate as a threshold to compare the difference in impact factors and citations between articles with higher reporting quality and others reporting relatively lower. Articles without IF or citations would not be included in the analysis. Bonferroni’s correction was applied to reduce the chance of type I error in mutiple comparisons. After a Bonferroni correction, a *p-value* less than 0.0021 (0.05/24) was considered as statistically significant difference. These statistical analyses were performed on SPSS v26.0 (IBM Corp., Armonk, NY, USA). ITSA was performed on python 3.10.7, “Statsmodels” was used for analysis, “Matplotlib” was used for creating the graph.

## Results

### Screening results and characteristics of included studies

A total of 5824 articles were identified, and 187 articles were finally included after the screening (Fig. 1). Cohen’s kappa index between the two reviewers (R.Z. and W.Z.) in the final dicision of inclusion is 0.69, indicating a relatively consistent level. The complete list of included articles is available in S2 File. The number of articles has been on the rise in recent years. Articles from the USA were at most (56,29.9%). Cancer (32,16.7%) and Diabetes Mellitus (22,11.5%) are the most studied diseases. The most common therapy is drug (106,56.7%). Most studies used a single database (121, 64.7%), with multicenter registries accounting for the majority (116, 62%). Details about the characteristics of included studies are shown in Table 1.

### Reporting quality of cohort studies using RWD

The mean ± SD of the percentage of adequately reported items in the 187 articles was 44.7 ± 14.3 with a range of 11.1–87% (disregarding inapplicable items). 72 articles (38.5%) adequately reported 50% and above items, the evaluation details of each article can be obtained in S3 File.

Out of 23 items in total, the adequate reporting rate of 10 items reached 50%, and the reporting rate of some vital items was inadequate. Of 66 (35.3%) studies involving database linkage, 33 (50%), 12 (18.2%), and 28(42.4%) studies adequately reported databases linkage in title or abstract (R1.3), flowcharts or other diagrams to display linkage (R6.3), and the detail of linkage (R12.3) respectively (Table 2). Of the 75 (40.1%) articles that adequately reported codes or algorithms of population selection (R6.1), only 21(28%), 19 (25.3%) articles adequately reported the validation methods (R6.2), and the limitations of codes or algorithms and the validation used to population selection (R19.1c) respectively (Table 2).

In addition, the reporting of some other items was also critically insufficient, such as codes of algorithms (R7.1a:28, 15%; R7.1b:43, 23%; R7.1c:23, 12.3%), data-cleaning methods (R12.2:65, 34.8%), discussion of change in eligibility of results over time (R19.1e:34, 18.2%), and availability of study protocol and raw study (R22.1a:44, 23.5%, R22.1b:38, 20.3%) (Table 2). Interrater agreement in evalutaion was substantial (kappa = 0.79).

### Reporting before and after the release of RECORD

Compared to Pre-RECORD period (33 articles), significant improvement in reporting quality for only one items in Post-RECORD period (116 articles): reporting of availability of raw data (R22.1b, *p<0.001*= (Table 3). There was no significant improvement in the overall report quality.

### ITSA of adequate reporting rate

The ITSA result showed that there was no significant change in the slope before and after the release of RECORD (Oct,2015) (coefficient = -0.003, standard error = 0.004, *p = 0.42*), the same goes for level change (coefficient = 0.006, standard error = 0.79, *p = 0.12*) (Fig. 2).

### Correlations of journal IFs and citations with reporting quality

The journal IFs were significantly higher for adequately reported articles in 6 items: population selection methods (R6.1: *p = 0.04*), codes or algorithms of outcomes (R7.1b: *p = 0.014*), the extent of database accessed (R12.1: *p = 0.004*), eligibility of results over time (R19.1e: *p = 0.001*), availability of raw data and supplementary materials (R22.1b: *p = 0.022*; and R22.1c: *p<0.001*=(Table 4). But only item R19.1e and R22.1c still have significant differences after Bonferroni’s correction. The citations of articles were also significantly higher for adequately reported articles in 6 items: R6.1: *p = 0.035*; R7.1a: *p = 0.02*; R7.1c: *p = 0.001*; R19.1a: *p = 0.01*; R19.1d: *p = 0.001*; R22.1b: *p = 0.015* (Table 4). Only item R7.1c and R19.1d still have significant differences after Bonferroni’s correction .In total, the journal IFs was significantly higher for articles with advanced reporting rates (≥ 50%)(IFs: 4.2 *versus* 3.17, *p = 0.002*), and there is no significant difference after Bonferroni’s correction in citations(Table 4).

## Discussion

To our knowledge, this is the first time that the complete RECORD checklist was used to evaluate the reporting quality of studies using RWD, with the analysis of the change in reporting quality and its relationship to the journal IFs and citations. We found that only 72 (38.5%) articles adequately reported more than 50% of the items, and some vital items were very insufficiently reported. The overall reporting quality was poor. Some items were reported similarly to analogous studies, such as the codes and algorithms for population selection and their validation studies, as well as data cleaning (R6.1, R6.2, R12.2), while some items were reported worse than analogous studies, such as the codes and algorithms of exposures, outcomes, confounders, and the availability of supplementary materials (R7.1a, R7.1b, R22.1c) [19, 25]. While most studies stated that there were inherent limitations when using RWD, there is insufficient discussion of the limitations in some specific areas. Data cleaning, data linkage, and disclosure of relevant information were also severely underreported.

Previous studies have investigated changes in reporting quality before and after the release of other checklists, such as STROBE [26], CONSORT [27], etc. We used similar approaches to compare changes in reporting quality before and after the release of RECORD, and we found statistically significant increases in reporting quality for only one item which suggests years after the release of RECORD, the reporting quality of cohort studies has not improved in total. ITSA analysis also shows that the release of RECORD has little effect on the improvement of reporting quality.

The journal IFs were related to some areas and significantly higher in high-reporting quality articles. In addition, high-reporting quality articles had higher citations than low report quality articles, but there was no longer a significant difference after Bonferroni’s correction. Nevertheless, our results were compatible with the study conducted by Pol CB van der et al. that high-quality studies were cited more frequently [28]. Interestingly, we found that few high-quality articles had low journal IFs and few citations instead, and vice versa. However, our analysis was cursory because the effect of time was not removed, and the newly published articles may have fewer citations.

RWE, on one hand, holds the potential to address important questions [29], on the other hand, there is some controversy due to the issues such as the quality of data sources or studies. The reporting quality issue of concern to this research, to some extent, influences the quality of RWE and its utility in decision-making. Databases may have issues with incomplete, inconsistent, and inaccurate coding, which makes it more challenging and complex to create data linkages and seriously impedes the reproducibility and replicability of studies, and therefore distinct reporting of codes or algorithms and their validation allows critical assessment by readers and benefits the generalization of study findings [30–32]. Linkage of databases can supplement and enrich data sources and transparent reporting can increase confidence in RWE [33, 34]. Only 28 (42.4%) articles in the present research reported details such as the level or methods of linkage, yet this may still be insufficient, and some extended guidelines have been developed for more detailed issues of data linkage [34, 35]. The disclosure of research-related information is also fundamental to improving the transparency of studies, especially the availability of raw data, which enables readers to assess the authenticity and reliability of the findings.

We believe that establishing a high-quality analytical database with accuracy, completeness, consistency, and wide applicability is the core of acquiring reliable RWE [36], and the crucial elements involve codes or algorithms and their validations, quality of data linkage, data cleaning, and the establishment of data specifications. This necessitates that we concentrate on both the quality of the research process, including methodology and reporting quality, as well as the quality of the data sources, such as standardized data structures and rigorous data quality assessments [37, 38]. In the meantime, health data not collected for specific purposes are generally not standardized, in contrast to the strictly conducted RCTs. It is almost impossible to balance all confounders and eliminate the impact of quality problems such as data errors and missing. However, we must still be comprehensively aware of the specific limitations of studies using such data and do everything possible to lessen their impact.

Our research demonstrated the current reporting state of cohort studies using RWD in recent years. There are no restrictions on population and exposure measures, and results have wider applicability. According to our research, we can recognize that there are various issues with this type of study which may be caused by the inadequate dissemination and endorsement of pertinent guidelines, the incomplete methodology consensus, etc. However, we realized that full compliance with the RECORD guidelines is almost impossible in some circumstances because of the technical issues involved in data processing, local policy implications, etc. But for real-world studies, RWD should be made realistic, standardized, and easy to handle from the inception of the study dataset development, otherwise such studies would create more risks of bias different from traditional research methods, which requires the Involvement of policy makers, technical personnel, investigators, clinicians, epidemiologists, and methodologists. And at least, researchers can standardize data and research processes as much as possible, we anticipate that our research can promote the spreading of RECORD and suggest the possible direction for researchers to improve RWE quality.

There are some limitations or weaknesses to this research. First, we only used the RECORD checklist to evaluate included articles, and other important aspects of observational studies mentioned in the STROBE checklist were not evaluated, such as details of study design, statistical methods, and reporting of results. Second, some items may not have undergone a strict enough evaluation, such as item R12.3 was deemed sufficient if the author described the level, techniques, and methods of data link or the method to evaluate its quality, which did not demand to be fully detailed. Finally, our search strategy cannot retrieve studies that did not mention the “real world” in the paper, consequently, we may overlook many articles that met the criteria, but the final inclusion result was within the acceptable range.

To conclude, the endorsement of the RECORD checklist was generally inadequate in cohort studies using RWD and has not improved in recent years. Journal IFs and article citations were significantly related to the reporting of some areas. We encourage researchers to endorse relevant guidelines when utilizing RWD for research to maximize the value of RWD, obtain high-quality RWE, and prevent misleading clinical decisions.

## Electronic supplementary material

Below is the link to the electronic supplementary material.

Supplementary Material 1: Search strategy

Supplementary Material 2: Transformed RECORD checklist.

Supplementary Material 3: List of included articles.

Supplementary Material 4: Details of evaluation results.

## Data Availability

The full data set is available on request from the corresponding author.

## References

[1] Khozin S, Blumenthal GM, Pazdur R. Real-world data for clinical evidence generation in Oncology. J Natl Cancer Inst. 2017;109. 10.1093/jnci/djx187. 10.1093/jnci/djx187 29059439

[2] Gliklich RE, Leavy MB (2020).

[3] US Food and Drug Administration, Real-World Evidence. FDA 2022. https://www.fda.gov/science-research/science-and-research-special-topics/real-world-evidence (accessed November 1, 2022).

[4] Eichler H-G, Pignatti F, Schwarzer-Daum B, Hidalgo-Simon A, Eichler I, Arlett P (2021). Randomized controlled trials Versus Real World evidence: neither Magic nor myth. Clin Pharmacol Ther.

[5] Breckenridge AM, Breckenridge RA, Peck CC (2019). Report on the current status of the use of real-world data (RWD) and real-world evidence (RWE) in drug development and regulation. Br J Clin Pharmacol.

[6] Thompson D (2021).

[7] Raphael MJ, Gyawali B, Booth CM (2020). Real-world evidence and regulatory drug approval. Nat Rev Clin Oncol.

[8] Wang SV, Sreedhara SK, Schneeweiss S, REPEAT Initiative (2022). Reproducibility of real-world evidence studies using clinical practice data to inform regulatory and coverage decisions. Nat Commun.

[9] Wang SV, Schneeweiss S, Berger ML, Brown J, de Vries F, Douglas I (2017). Reporting to improve reproducibility and facilitate Validity Assessment for Healthcare Database Studies V1.0. Value Health J Int Soc Pharmacoeconomics Outcomes Res.

[10] Benchimol EI, Manuel DG, To T, Griffiths AM, Rabeneck L, Guttmann A (2011). Development and use of reporting guidelines for assessing the quality of validation studies of health administrative data. J Clin Epidemiol.

[11] Malone DC, Brown M, Hurwitz JT, Peters L, Graff JS (2018). Real-world evidence: useful in the Real World of US payer decision making? How? When? And what studies?. Value Health J Int Soc Pharmacoeconomics Outcomes Res.

[12] Benchimol EI, Smeeth L, Guttmann A, Harron K, Moher D, Petersen I (2015). The REporting of studies conducted using Observational routinely-collected health data (RECORD) statement. PLoS Med.

[13] Langan SM, Schmidt SA, Wing K, Ehrenstein V, Nicholls SG, Filion KB (2018). The reporting of studies conducted using observational routinely collected health data statement for pharmacoepidemiology (RECORD-PE). BMJ.

[14] Public Policy Committee, International Society of Pharmacoepidemiology (2016). Guidelines for good pharmacoepidemiology practice (GPP). Pharmacoepidemiol Drug Saf.

[15] The European Network of Centres for Pharmacoepidemiology and Pharmacovigilance (ENCePP). ENCePP Home Page n.d. https://www.encepp.eu/standards_and_guidances/ (accessed November 1, 2022).

[16] von Elm E, Altman DG, Egger M, Pocock SJ, Gøtzsche PC, Vandenbroucke JP (2014). The strengthening the reporting of Observational Studies in Epidemiology (STROBE) Statement: guidelines for reporting observational studies. Int J Surg Lond Engl.

[17] Pouwels KB, Widyakusuma NN, Groenwold RHH, Hak E (2016). Quality of reporting of confounding remained suboptimal after the STROBE guideline. J Clin Epidemiol.

[18] Antwi E, Amoakoh-Coleman M, Vieira DL, Madhavaram S, Koram KA, Grobbee DE (2020). Systematic review of prediction models for gestational hypertension and preeclampsia. PLoS ONE.

[19] Hemkens LG, Benchimol EI, Langan SM, Briel M, Kasenda B, Januel J-M (2016). The reporting of studies using routinely collected health data was often insufficient. J Clin Epidemiol.

[20] Wang X, Kattan MW (2020). Cohort studies: design, analysis, and reporting. Chest.

[21] Euser AM, Zoccali C, Jager KJ, Dekker FW (2009). Cohort studies: prospective versus retrospective. Nephron Clin Pract.

[22] Na D, Sr T, D O MB. Why observational studies should be among the tools used in comparative effectiveness research. Health Aff Proj Hope. 2010;29. 10.1377/hlthaff.2010.0666. 10.1377/hlthaff.2010.0666 20921481

[23] Tepe G, Zeller T, Moscovic M, Corpataux J-M, Christensen JK, Keirse K (2020). Paclitaxel-Coated Balloon for the treatment of Infrainguinal Disease: 12-Month Outcomes in the All-Comers Cohort of BIOLUX P-III Global Registry. J Endovasc Ther Off J Int Soc Endovasc Spec.

[24] Scottish Intercollegiate Guidelines Network. Search filters n.d. https://www.sign.ac.uk/what-we-do/methodology/search-filters/ (accessed November 1, 2022).

[25] Yolcu Y, Wahood W, Alvi MA, Kerezoudis P, Habermann EB, Bydon M (2020). Reporting methodology of Neurosurgical Studies utilizing the American College of Surgeons-National Surgical Quality Improvement Program Database: a systematic review and critical Appraisal. Neurosurgery.

[26] Rao A, Brück K, Methven S, Evans R, Stel VS, Jager KJ (2016). Quality of reporting and Study Design of CKD Cohort Studies assessing mortality in the Elderly before and after STROBE: a systematic review. PLoS ONE.

[27] Plint AC, Moher D, Morrison A, Schulz K, Altman DG, Hill C (2006). Does the CONSORT checklist improve the quality of reports of randomised controlled trials? A systematic review. Med J Aust.

[28] van der Pol CB, McInnes MDF, Petrcich W, Tunis AS, Hanna R (2015). Is quality and completeness of reporting of systematic reviews and Meta-analyses published in high impact Radiology Journals Associated with Citation Rates?. PLoS ONE.

[29] Penberthy LT, Rivera DR, Lund JL, Bruno MA, Meyer A-M (2022). An overview of real-world data sources for oncology and considerations for research. CA Cancer J Clin.

[30] Carter B, Verity Bennett C, Bethel J, Jones HM, Wang T, Kemp A (2019). Identifying cerebral palsy from routinely-collected data in England and Wales. Clin Epidemiol.

[31] Lyu H, Haider A, Landman A, Raut C (2019). The Opportunities and Shortcomings of using Big Data and National Databases for Sarcoma Research. Cancer.

[32] Twiss E, Krijnen P, Schipper I (2021). Accuracy and reliability of injury coding in the national Dutch Trauma Registry. Int J Qual Health Care J Int Soc Qual Health Care.

[33] Rivera DR, Gokhale MN, Reynolds MW, Andrews EB, Chun D, Haynes K (2020). Linking electronic health data in pharmacoepidemiology: appropriateness and feasibility. Pharmacoepidemiol Drug Saf.

[34] Pratt NL, Mack CD, Meyer AM, Davis KJ, Hammill BG, Hampp C (2020). Data linkage in pharmacoepidemiology: a call for rigorous evaluation and reporting. Pharmacoepidemiol Drug Saf.

[35] Gilbert R, Lafferty R, Hagger-Johnson G, Harron K, Zhang L-C, Smith P (2018). GUILD: GUidance for information about linking data sets. J Public Health Oxf Engl.

[36] Ehsani-Moghaddam B, Martin K, Queenan JA (2021). Data quality in healthcare: a report of practical experience with the canadian primary care Sentinel Surveillance Network data. Health Inf Manag J.

[37] Reps JM, Schuemie MJ, Suchard MA, Ryan PB, Rijnbeek PR (2018). Design and implementation of a standardized framework to generate and evaluate patient-level prediction models using observational healthcare data. J Am Med Inform Assoc JAMIA.

[38] Blacketer C, Defalco FJ, Ryan PB, Rijnbeek PR (2021). Increasing trust in real-world evidence through evaluation of observational data quality. J Am Med Inform Assoc JAMIA.

