# Supplementary material for: Evaluation of reporting quality of cohort studies using real-world data based on RECORD: systematic review
Source: BMC Med Res Methodol. 2023 Jun 29;23:152. doi: 10.1186/s12874-023-01960-2 (PMC10308622; doi:10.1186/s12874-023-01960-2)
Supplement: Supplementary file 1 — Supplementary Material 1: Search strategy [file 12874_2023_1960_MOESM1_ESM.docx]

**S1 File. Search strategy.**

**Databases**: Embase and MEDLINE in OVID

**Search date**: April 29, 2022

Cohort studies：

1. Epidemiologic studies/
2. exp cohort studies/
3. (cohort adj4 (study or studies)).pt,tw.
4. (observational adj4 (study or studies)). pt,tw.
5. Longitudinal. pt,tw.
6. Retrospective. pt,tw.
7. Prospective. pt,tw.
8. Group*.pt,tw.
9. or/1-8

Data source:

1. routinely collected data.tw.
2. routinely collected health data.tw.
3. Health information system.tw.
4. Hospital information system.tw.
5. HIS.tw.
6. Electronic health record.tw.
7. EHR.tw.
8. Electronic medical record.tw.
9. EMR.tw.
10. electronic patient record.tw.
11. electronic healthcare record.tw.
12. Medical record.tw.
13. patient record.tw.
14. patient medical record.tw.
15. PMR.tw.
16. Patient health record.tw.
17. PHR.tw.
18. health record.tw.
19. healthcare record.tw.
20. health care database.tw.
21. healthcare data.tw.
22. national database.tw.
23. medical database.tw.
24. Registries.tw.
25. register*.tw.
26. registr*.tw.
27. Insurance records.tw.
28. Insurance claims.tw.
29. Insurance data.tw.
30. Pharmacy record.tw.
31. Wearable*.tw.
32. Or/10-40

Efficacy/safety：

1. Effect*.tw.
2. efficacy.tw.
3. Effective*.tw.
4. Valid*.tw.
5. Safety.tw.
6. Adverse reaction*.tw.
7. ADR*.tw.
8. or/42-48\
9. real world
10. 9 and 41 and 49 and 50

Excluded publication types or article types：

1. Review.pt,tw.
2. Biography.pt.
3. Bibliography.pt.
4. Autobiography.pt.
5. Case Reports.pt,tw.
6. Clinical Conference.pt.
7. Protocol.pt,tw.
8. Comment.pt,tw.
9. Consensus Development Conference.pt.
10. Editorial.pt.
11. Letter.pt,tw.
12. Directory.pt,tw.
13. Meta-Analysis.tw.
14. Systematic review.tw.
15. Guideline.pt,tw.
16. Practice Guideline.pt,tw.
17. or/52-67

Excluded outcomes:

1. Economics/
2. Cost allocation/
3. "costs and cost analysis"/
4. Cost-benefit analysis/
5. Cost control/
6. Cost savings/
7. Cost of illness/
8. Cost sharing/
9. "deductibles and coinsurance"/
10. Medical savings accounts/
11. Health care costs/
12. Direct service costs/
13. Drug costs/
14. Employer health costs/
15. Hospital costs/
16. Health expenditures/
17. Capital expenditures/
18. Value of life/
19. exp economics, hospital/
20. exp economics, medical/
21. Economics, nursing/
22. Economics, pharmaceutical/
23. exp "fees and charges"/
24. exp budgets/
25. (low adj3 cost).mp.
26. (high adj3 cost).mp.
27. (health?care adj cost$).mp.
28. (fiscal or funding or financial or finance).tw.
29. (cost adj3 variable).mp.
30. (cost adj3 estimate$).mp.
31. (unit adj3 cost$).mp.
32. (economic$ or pharmacoeconomic$ or price$ or pricing).tw.
33. or/69-100
34. 51 not (68 or 101)
35. limit 102 to humans
36. limit 103 to English
37. limit 104 to yr="2013 - 2021"
