# Supplementary material for: Evaluation of reporting quality of cohort studies using real-world data based on RECORD: systematic review
Source: BMC Med Res Methodol. 2023 Jun 29;23:152. doi: 10.1186/s12874-023-01960-2 (PMC10308622; doi:10.1186/s12874-023-01960-2)
Supplement: Supplementary file 2 — Supplementary Material 2: Transformed RECORD checklist. [file 12874_2023_1960_MOESM2_ESM.docx]

**S1 Table. Transformed RECORD^a^ checklist with descriptions and examples.**

| Items | Questions, Descriptions, and Examples |
| --- | --- |
| Title and abstract | |
| R1.1a | Was the type of data used named in the title and abstract? |
| R1.1b | Were the names of the databases reported in the title and abstract? |
| R1.2a | Was the geographic region within which the study took place reported in the title and abstract? |
| R1.2b | Was the time frame within which the study took reported in the title and abstract? |
|  | **Description:** For multicenter studies, only the name of the pooled database was required, and the geographic region was not equivalent to the author's region.  ***Example:*** *The Swedish National Register for Systemic Treatment of Psoriasis (PsoReg) was established in 2006. This article analyzes the implementation phase of biologics in the treatment of moderate to severe psoriasis in Sweden in the period 2006–2012 [1].* |
| R1.3 | Was the conducted linkage between databases for the study stated clearly in the title and abstract (if applicable)? |
|  | **Description:** Studies that did not involve the linkage of databases, such as when the data source was a single center or a registry that used a uniform data collection and standardized methodology, were not applicable to this item.  ***Example:*** *This retrospective administrative claims-based study utilized medical, pharmacy, and enrollment data linked to laboratory results information from a large United States health plan (January 1, 2006, to March 31, 2011) and included individuals with recorded evidence of T2DM and HCh [2].* |
| Methods | |
| R6.1 | Were the methods of study population selection (such as codes or algorithms used to identify subjects) listed in detail? |
|  | **Description:** This item was considered partly reported when the codes or algorithms for population selection did not provide. Reporting is also considered adequate if the author stated that the codes or algorithms were used and provided a reasonable explanation for why they cannot be disclosed.  ***Example:*** *Data on morbidity were obtained from the National Patient Register, where diagnoses from all hospital admissions (from 1978) and outpatient activities (from 1995) are listed according to the International Classification of Diseases (ICD) codes (ICD-8: 1978–1993; ICD-10: 1994–2009). This register also includes information on surgical procedures and other non-trivial treatments coded by use of SKS (a Danish coding system) … patients with RA were identified by ICD-10 codes M05–M06 in combination with dispensed disease-modifying antirheumatic drugs (DMARDs) within 1 year before or after the time of diagnosis [3].* |
| R6.2 | For the codes or algorithms used to select the population, were the validation studies referenced or detailed methods and results provided if the validation was conducted for this study and not published elsewhere (if applicable)? |
|  | **Description:** Studies that adequately reported the codes or algorithms for population selection were applicable to this item.  ***Examples:*** *1. Sabidó M et al. cited the validation study of diagnostic coding [4].*  *2. A manual chart review of 20 randomly selected non-overlapping subjects was performed using a standardized adjudication form (Online Figure 1). Based on this review, the algorithm correctly identified all 20 subjects [5].* |
| R6.3 | Was the flow diagram or other graphical display to demonstrate the data linkage process used (if applicable)? |
|  | **Description:** Not applicable same as R1.3.  ***Example:*** *Weissler EH et al. used a flowchart to display the linkage between NCDR registry collected data and CMS data [6].* |
| R7.1a | Was a complete list of the codes and algorithms used to classify exposures provided? |
| R7.1b | Was a complete list of the codes and algorithms used to classify outcomes provided? |
| R7.1c | Was a complete list of the codes and algorithms used to classify confounders and effect modifiers provided? |
|  | **Description:** Reporting is also considered adequate if the author stated that the codes or algorithms were used and provided a reasonable explanation for why they cannot be disclosed.  ***Example:*** *The study outcome was the combined endpoint of MI (ICD-10: I21–I22), stroke (ICD-10: I60, I61, I63 and I64) or cardiovascular death (ICD-10 code: I00–I99 listed as the primary cause of death) …NSAID use was determined …: ibuprofen (ATC code: M01AE01), diclofenac(M01AB05), etodolac (M01AB08), celecoxib (M01AH01), piroxicam (M01AC01), rofecoxib (M01AH02), naproxen (M01AE02), ketoprofen (M01AE03), nabumetone (M01AX01) and indometacin (M01AB01) … details on ATC and ICD codes used in the study are listed in online supplementary table S1 [3].* |
| R12.1 | Did the authors describe the extent to which the investigators had access to the database used to create the study population? |
|  | ***Example:*** *From January 2012 until December 2016, 2,137 patients were hospitalized for STEMI in our center [7].* |
| R12.2 | Did the authors provide information on the data-cleaning methods used in the study? |
|  | ***Example:*** *Once the data were documented in the pCRFs by the first research staff, the pCRFs were randomly reviewed and corrected by the second research staff to ensure accuracy and completeness. Afterwards, an independent double data entry and validation process for the pCRFs was conducted using the Epidata® programme. Prior to analysis, the data discrepancy in the database was verified and reconciled [8].* |
| R12.3 | Did the authors state whether the study included person-level, institutional-level, or other data linkage across two or more databases and provide the linkage techniques and methods used to evaluate linkage quality? |
|  | **Description:** Not applicable same as R1.3.  ***Example:*** *we linked GWTG-Stroke data to Medicare claims by matching on a series of indirect identifiers…for the assessment of longitudinal outcomes. This linkage method, developed by our analytic team, has been successfully completed and validated using Medicare inpatient claims. Previous work has shown that patients in the linked GWTG-Stroke/CMS database are representative of the national Medicare ischemic stroke population. Quintiles Inc serves as the data collection (through its patient management tool) and coordination center for GWTG-Stroke [9].* |

**Continued.**

| Items | Questions, Descriptions, and Examples |
| --- | --- |
| Results | |
| R13.1 | Did the authors describe the selection of the persons included in the study in detail, including the number and reasons for filtering? |
|  | ***Example:*** *Racey CS et al. used a flowchart to display the number of people screened and the reason for each step for population screening [10].* |
| Discussion | |
| R19.1a | Did the authors declare that there are inherent limitations when using data that were not created or collected to answer the specific research question(s)? |
| R19.1b | Did the authors discuss the risk of misclassification and the potential impacts on study findings? |
| R19.1c | Did the authors discuss the limitations of the codes/algorithms (for population selection) and the validation studies (if applicable)? |
| R19.1d | Did the authors discuss the implications of the missing variables, unmeasured confounding, or missing data? |
| R19.1e | Did the authors discuss the changes in the eligibility of results over time? |
|  | **Description:** Studies that adequately reported the codes or algorithms for population selection were applicable to item R19.1c  ***Examples:*** *1. Our study has several limitations common to administrative databases. Residual confounding could account for some of the observed associations …coding inaccuracies will undoubtedly occur and can affect our estimates. However, several reports have demonstrated that coding for aneurysm and cerebrovascular disease has shown nearly perfect association with medical record review. Although SPARCS includes all hospitals from the entire New York State, the generalization of this analysis to the entire US population is uncertain. SPARCS does not provide any clinical information on the structure, size, or location of the aneurysms, which are important factors in cerebrovascular neurosurgery …we were lacking post-hospitalization, and long-term data on our patients [11].*  *2. In the current registry, over time, patient numbers for some parameters (especially e.g. testosterone, prostate volume, and S-ALP where recording was optional) became quite small in some patient subgroups (e.g. metastatic patients) which may affect the reliability of measurements towards the end of the observation period in these cases [12].* |
| Other information | |
| R22.1a | Did the authors provide information on how to access the study protocol? |
| R22.1b | Did the authors provide information on how to access the raw data? |
| R22.1c | Did the authors provide information on how to access other supplementary material? |
|  | ***Example:*** *DeMichele A et al. reported the method of obtaining the study protocol, raw data and supplementary materials [13].* |

^a^RECORD, the REporting of studies Conducted using Observational Routinely-collected health Data statement

References

1. Norlin JM, Carlsson KS, Persson U, Schmitt-Egenolf M. Register-Based Evaluation of Relative Effectiveness of New Therapies: Biologics Versus Conventional Agents in Treatment of Psoriasis in Sweden. BioDrugs. 2015;29(6):389-398. doi:10.1007/s40259-015-0151-4
2. Swindle JP, Ye X, Mallick R, Song R, Horstman T, Bays HE. Colesevelam, Ezetimibe, and Patients With Type 2 Diabetes Mellitus: Characteristics and Clinical Outcomes From a Health Care Database. Ann Pharmacother. 2014;48(7):847-855. doi:10.1177/1060028014531737
3. Lindhardsen J, Gislason GH, Jacobsen S, et al. Non-steroidal anti-inflammatory drugs and risk of cardiovascular disease in patients with rheumatoid arthritis: a nationwide cohort study. Ann Rheum Dis. 2014;73(8):1515-1521. doi:10.1136/annrheumdis-2012-203137
4. Sabidó M, Hohenberger T, Grassi G. Pharmacological intervention in hypertension using beta-blockers: Real-world evidence for long-term effectiveness. Pharmacol Res. 2018;130:191-197. doi:10.1016/j.phrs.2018.01.010
5. Bottinor WJ, Shuey MM, Manouchehri A, et al. Renin-Angiotensin-Aldosterone System Modulates Blood Pressure Response During Vascular Endothelial Growth Factor Receptor Inhibition. JACC CardioOncol. 2019;1(1):14-23. doi:10.1016/j.jaccao.2019.07.002
6. Bottinor WJ, Shuey MM, Manouchehri A, et al. Renin-Angiotensin-Aldosterone System Modulates Blood Pressure Response During Vascular Endothelial Growth Factor Receptor Inhibition. JACC CardioOncol. 2019;1(1):14-23. doi:10.1016/j.jaccao.2019.07.002
7. Desperak P, Hawranek M, Chodór PA, et al. Comparison of the everolimus-eluting bioresorbable vascular scaffold versus the everolimus-eluting metallic stent in real-world patients with ST-segment elevation myocardial infarction. Postepy Kardiol Interwencyjnej. 2020;16(1):49-57. doi:10.5114/aic.2020.93912
8. Sangroongruangsri S, Chaikledkaew U, Kumluang S, et al. Real-World Safety of Intravitreal Bevacizumab and Ranibizumab Treatments for Retinal Diseases in Thailand: A Prospective Observational Study [published correction appears in Clin Drug Investig. 2019 Jan 19;:]. Clin Drug Investig. 2018;38(9):853-865. doi:10.1007/s40261-018-0678-5
9. Sangroongruangsri S, Chaikledkaew U, Kumluang S, et al. Real-World Safety of Intravitreal Bevacizumab and Ranibizumab Treatments for Retinal Diseases in Thailand: A Prospective Observational Study [published correction appears in Clin Drug Investig. 2019 Jan 19;:]. Clin Drug Investig. 2018;38(9):853-865. doi:10.1007/s40261-018-0678-5
10. Racey CS, Albert A, Donken R, et al. Cervical Intraepithelial Neoplasia Rates in British Columbia Women: A Population-Level Data Linkage Evaluation of the School-Based HPV Immunization Program. J Infect Dis. 2020;221(1):81-90. doi:10.1093/infdis/jiz422
11. Bekelis K, Missios S, Coy S, Rahmani R, Singer RJ, MacKenzie TA. Surgical Clipping versus Endovascular Intervention for the Treatment of Subarachnoid Hemorrhage Patients in New York State. PLoS One. 2015;10(9):e0137946. Published 2015 Sep 11. doi:10.1371/journal.pone.0137946
12. Geiges G, Harms T, Rodemer G, et al. Degarelix therapy for prostate cancer in a real-world setting: experience from the German IQUO (Association for Uro-Oncological Quality Assurance) Firmagon® registry. BMC Urol. 2015;15:122. Published 2015 Dec 16. doi:10.1186/s12894-015-0116-4
13. DeMichele A, Cristofanilli M, Brufsky A, et al. Comparative effectiveness of first-line palbociclib plus letrozole versus letrozole alone for HR+/HER2- metastatic breast cancer in US real-world clinical practice. Breast Cancer Res. 2021;23(1):37. Published 2021 Mar 24. doi:10.1186/s13058-021-01409-8
