# Supplementary material for: Evaluation of reporting quality of cohort studies using real-world data based on RECORD: systematic review
Source: BMC Med Res Methodol. 2023 Jun 29;23:152. doi: 10.1186/s12874-023-01960-2 (PMC10308622; doi:10.1186/s12874-023-01960-2)
Supplement: Supplementary file 3 — Supplementary Material 3: List of included articles. [file 12874_2023_1960_MOESM3_ESM.docx]

**S2 File. List of included articles**

1. Kübler P, Jankowska EA, Ferenc M, Ponikowski P, Banasiak W, Reczuch K. Comparison of drug-eluting stents to bare-metal stents in ST-elevation myocardial infarction in long-term follow-up. Kardiol Pol. 2013;71(1):25-31.
2. Chambost H, Santagostino E, Laffan M, Kavakli K; ONE Registry Steering Committee on behalf of the investigators. Real-world outcomes with recombinant factor VIIa treatment of acute bleeds in haemophilia patients with inhibitors: results from the international ONE registry. Haemophilia. 2013;19(4):571-577. doi:10.1111/hae.12140
3. Harrison MR, George DJ, Walker MS, et al. ＂Real world＂ treatment of metastatic renal cell carcinoma in a joint community-academic cohort: progression-free survival over three lines of therapy. Clin Genitourin Cancer. 2013;11(4):441-450. doi:10.1016/j.clgc.2013.05.002
4. Gitt AK, Bramlage P, Binz C, Krekler M, Deeg E, Tschöpe D. Prognostic implications of DPP-4 inhibitor vs. sulfonylurea use on top of metformin in a real world setting - results of the 1 year follow-up of the prospective DiaRegis registry. Int J Clin Pract. 2013;67(10):1005-1014. doi:10.1111/ijcp.12179
5. Belperio PS, Hwang EW, Thomas IC, Mole LA, Cheung RC, Backus LI. Early virologic responses and hematologic safety of direct-acting antiviral therapies in veterans with chronic hepatitis C. Clin Gastroenterol Hepatol. 2013;11(8):1021-1027. doi:10.1016/j.cgh.2013.03.006
6. Larsen TB, Rasmussen LH, Skjøth F, et al. Efficacy and safety of dabigatran etexilate and warfarin in "real-world" patients with atrial fibrillation: a prospective nationwide cohort study. J Am Coll Cardiol. 2013;61(22):2264-2273. doi:10.1016/j.jacc.2013.03.020
7. Park KW, Lee JM, Kang SH, et al. Safety and efficacy of second-generation everolimus-eluting Xience V stents versus zotarolimus-eluting resolute stents in real-world practice: patient-related and stent-related outcomes from the multicenter prospective EXCELLENT and RESOLUTE-Korea registries. J Am Coll Cardiol. 2013;61(5):536-544. doi:10.1016/j.jacc.2012.11.015
8. Park KW, et al, Everolimus- versus sirolimus-eluting stents for the treatment of unprotected left main coronary artery stenosis (results from the EXCELLENT registry), Int J Cardiol (2013), <http://dx.doi.org/10.1016/j.ijcard.2013.03.086>
9. Rubboli A, Saia F, Sciahbasi A, et al. Outcome of patients on oral anticoagulation undergoing coronary artery stenting: data from discharge to 12 months in the Warfarin and Coronary Stenting (WAR-STENT) Registry. J Invasive Cardiol. 2014;26(11):563-569.
10. Bertin P, Taieb C. NSAID-sparing effect of glucosamine hydrochloride in patients with knee osteoarthritis: an analysis of data from a French database. Curr Med Res Opin. 2014;30(2):271-277. doi:10.1185/03007995.2013.855184
11. Swindle JP, Ye X, Mallick R, Song R, Horstman T, Bays HE. Colesevelam, Ezetimibe, and Patients With Type 2 Diabetes Mellitus: Characteristics and Clinical Outcomes From a Health Care Database. Ann Pharmacother. 2014;48(7):847-855. doi:10.1177/1060028014531737
12. Lindhardsen J, Gislason GH, Jacobsen S, et al. Non-steroidal anti-inflammatory drugs and risk of cardiovascular disease in patients with rheumatoid arthritis: a nationwide cohort study. Ann Rheum Dis. 2014;73(8):1515-1521. doi:10.1136/annrheumdis-2012-203137
13. Tentzeris I, Rohla M, Jarai R, et al. Influence of high-dose highly efficient statins on short-term mortality in patients undergoing percutaneous coronary intervention with stenting for acute coronary syndromes. Am J Cardiol. 2014;113(7):1099-1104. doi:10.1016/j.amjcard.2013.12.012
14. Lee JM, Park KW, Han JK, et al. Three-year patient-related and stent-related outcomes of second-generation everolimus-eluting Xience V stents versus zotarolimus-eluting resolute stents in real-world practice (from the Multicenter Prospective EXCELLENT and RESOLUTE-Korea Registries). Am J Cardiol. 2014;114(9):1329-1338. doi:10.1016/j.amjcard.2014.07.065
15. Larsen TB, Rasmussen LH, Gorst-Rasmussen A, et al. Myocardial ischemic events in 'real world' patients with atrial fibrillation treated with dabigatran or warfarin. Am J Med. 2014;127(4):329-336.e4. doi:10.1016/j.amjmed.2013.12.005
16. Pasternak B, Svanström H, Melbye M, Hviid A. Association of treatment with carvedilol vs metoprolol succinate and mortality in patients with heart failure [published correction appears in JAMA Intern Med. 2014 Nov;174(11):1875]. JAMA Intern Med. 2014;174(10):1597-1604. doi:10.1001/jamainternmed.2014.3258
17. Hatoum H, Khanna D, Lin SJ, Akhras KS, Shiozawa A, Khanna P. Achieving serum urate goal: a comparative effectiveness study between allopurinol and febuxostat. Postgrad Med. 2014;126(2):65-75. doi:10.3810/pgm.2014.03.2741
18. Wijeysundera HC, Bennell MC, Qiu F, et al. Comparative-effectiveness of revascularization versus routine medical therapy for stable ischemic heart disease: a population-based study. J Gen Intern Med. 2014;29(7):1031-1039. doi:10.1007/s11606-014-2813-1
19. Hjalte F, Steen Carlsson K, Schmitt-Egenolf M. Real-world outcome analysis of continuously and intermittently treated patients with moderate to severe psoriasis after switching to a biologic agent. Dermatology. 2015;230(4):347-353. doi:10.1159/000371881
20. Xian Y, Wu J, O'Brien EC, et al. Real world effectiveness of warfarin among ischemic stroke patients with atrial fibrillation: observational analysis from Patient-Centered Research into Outcomes Stroke Patients Prefer and Effectiveness Research (PROSPER) study. BMJ. 2015;351:h3786. Published 2015 Jul 31. doi:10.1136/bmj.h3786
21. O'Brien EC, Greiner MA, Xian Y, et al. Clinical Effectiveness of Statin Therapy After Ischemic Stroke: Primary Results From the Statin Therapeutic Area of the Patient-Centered Research Into Outcomes Stroke Patients Prefer and Effectiveness Research (PROSPER) Study. Circulation. 2015;132(15):1404-1413. doi:10.1161/CIRCULATIONAHA.115.016183
22. Tan HS, Li H, Hong YW, et al. Efficacy and Safety of an Attenuated-Dose Sunitinib Regimen in Metastatic Renal Cell Carcinoma: Results From a Prospective Registry in Singapore. Clin Genitourin Cancer. 2015;13(4):e285-e295. doi:10.1016/j.clgc.2014.11.004
23. Motovska Z, Ondrakova M, Bednar F, Knot J, Ulman J, Maly M. Selection of P2Y12 antagonist, treatment initiation, and predictors of high on-treatment platelet reactivity in a "Real World" registry. Thromb Res. 2015;135(6):1093-1099. doi:10.1016/j.thromres.2015.04.014
24. Kim SB, Kok YT, Thuan TV, Chao TY, Shen ZZ. Safety Results of Docetaxel-(Taxotere®)-Based Chemotherapy in Early Breast Cancer Patients of Asia-Pacific Region: Asia-Pacific Breast Initiative II. J Breast Cancer. 2015;18(4):356-364. doi:10.4048/jbc.2015.18.4.356
25. Schmieder RE, Potthoff SA, Bramlage P, et al. Patients With Newly Diagnosed Hypertension Treated With the Renin Angiotensin Receptor Blocker Azilsartan Medoxomil vs Angiotensin-Converting Enzyme Inhibitors: The Prospective EARLY Registry. J Clin Hypertens (Greenwich). 2015;17(12):947-953. doi:10.1111/jch.12603
26. Bekelis K, Missios S, Coy S, Rahmani R, Singer RJ, MacKenzie TA. Surgical Clipping versus Endovascular Intervention for the Treatment of Subarachnoid Hemorrhage Patients in New York State. PLoS One. 2015;10(9):e0137946. Published 2015 Sep 11. doi:10.1371/journal.pone.0137946
27. Song PS, Song YB, Yang JH, et al. Triple versus dual antiplatelet therapy after percutaneous coronary intervention for coronary bifurcation lesions: results from the COBIS (COronary BIfurcation Stent) II Registry. Heart Vessels. 2015;30(4):458-468. doi:10.1007/s00380-014-0500-0
28. Kosty MP, Wozniak AJ, Jahanzeb M, et al. Effectiveness and safety of post-induction phase bevacizumab treatment for patients with non-small-cell lung cancer: results from the ARIES observational cohort study. Target Oncol. 2015;10(4):509-516. doi:10.1007/s11523-014-0355-4
29. Geiges G, Harms T, Rodemer G, et al. Degarelix therapy for prostate cancer in a real-world setting: experience from the German IQUO (Association for Uro-Oncological Quality Assurance) Firmagon® registry. BMC Urol. 2015;15:122. Published 2015 Dec 16. doi:10.1186/s12894-015-0116-4
30. Kern DM, Davis J, Williams SA, et al. Comparative effectiveness of budesonide/formoterol combination and fluticasone/salmeterol combination among chronic obstructive pulmonary disease patients new to controller treatment: a US administrative claims database study. Respir Res. 2015;16(1):52. Published 2015 Apr 23. doi:10.1186/s12931-015-0210-x
31. Gitt AK, Bramlage P, Schneider S, Tschöpe D. A real world comparison of sulfonylurea and insulin vs. incretin-based treatments in patients not controlled on prior metformin monotherapy. Cardiovasc Diabetol. 2015;14:13. Published 2015 Feb 3. doi:10.1186/s12933-015-0172-9
32. Norlin JM, Carlsson KS, Persson U, Schmitt-Egenolf M. Register-Based Evaluation of Relative Effectiveness of New Therapies: Biologics Versus Conventional Agents in Treatment of Psoriasis in Sweden. BioDrugs. 2015;29(6):389-398. doi:10.1007/s40259-015-0151-4
33. Kirsner RS, Sabolinski ML, Parsons NB, Skornicki M, Marston WA. Comparative effectiveness of a bioengineered living cellular construct vs. a dehydrated human amniotic membrane allograft for the treatment of diabetic foot ulcers in a real world setting. Wound Repair Regen. 2015;23(5):737-744. doi:10.1111/wrr.12332
34. Adogwa O, Elsamadicy AA, Han JL, Cheng J, Karikari I, Bagley CA. Do measures of surgical effectiveness at 1 year after lumbar spine surgery accurately predict 2-year outcomes?. J Neurosurg Spine. 2016;25(6):689-696. doi:10.3171/2015.8.SPINE15476
35. Imori Y, D'Ascenzo F, Gori T, et al. Impact of postdilatation on performance of bioresorbable vascular scaffolds in patients with acute coronary syndrome compared with everolimus-eluting stents: A propensity score-matched analysis from a multicenter "real-world" registry. Cardiol J. 2016;23(4):374-383. doi:10.5603/CJ.a2016.0052
36. Frisell T, Forsberg L, Nordin N, et al. Comparative analysis of first-year fingolimod and natalizumab drug discontinuation among Swedish patients with multiple sclerosis. Mult Scler. 2016;22(1):85-93. doi:10.1177/1352458515579216
37. Jiang B, He Y, Zuo F, et al. Effectiveness of varenicline and counselling for smoking cessation in an observational cohort study in China. BMJ Open. 2016;6(1):e009381. Published 2016 Jan 6. doi:10.1136/bmjopen-2015-009381
38. Saunders WB, Nguyen H, Kalsekar I. Real-world glycemic outcomes in patients with type 2 diabetes initiating exenatide once weekly and liraglutide once daily: a retrospective cohort study. Diabetes Metab Syndr Obes. 2016;9:217-223. Published 2016 Jul 15. doi:10.2147/DMSO.S103972
39. Welzel TM, Petersen J, Herzer K, et al. Daclatasvir plus sofosbuvir, with or without ribavirin, achieved high sustained virological response rates in patients with HCV infection and advanced liver disease in a real-world cohort [published correction appears in Gut. 2016 Dec;65(12 ):2060]. Gut. 2016;65(11):1861-1870. doi:10.1136/gutjnl-2016-312444
40. Lim YH, Lee Y, Shin J, et al. Comparisons of Clinical and Procedural Outcomes Between Transradial and Transfemoral Approaches in Percutaneous Coronary Intervention (from the Korean Transradial Intervention Prospective Registry). Am J Cardiol. 2016;117(8):1272-1281. doi:10.1016/j.amjcard.2016.01.020
41. McAdam-Marx C, Nguyen H, Schauerhamer MB, et al. Glycemic Control and Weight Outcomes for Exenatide Once Weekly Versus Liraglutide in Patients with Type 2 Diabetes: A 1-Year Retrospective Cohort Analysis. Clin Ther. 2016;38(12):2642-2651. doi:10.1016/j.clinthera.2016.11.003
42. Bardini G, Giannini S, Rotella CM, Pala L, Cresci B, Mannucci E. Lower and higher-potency statins on glycemic control in type 2 diabetes: A retrospective cohort study. Diabetes Res Clin Pract. 2016;120:104-110. doi:10.1016/j.diabres.2016.07.015
43. Kistner I, Zeymer U, Dechend R, et al. Benefits and Risks of Aliskiren Treatment in Patients With Type 2 Diabetes: Analyses of the 3A Registry. J Clin Hypertens (Greenwich). 2016;18(10):1045-1053. doi:10.1111/jch.12828
44. Zhang F, Yang J, Qian J, et al. Long-term performance of the second-generation cobalt-chromium sirolimus-eluting stents in real-world clinical practice: 3-year clinical outcomes from the prospective multicenter FOCUS registry. J Thorac Dis. 2016;8(7):1601-1610. doi:10.21037/jtd.2016.05.11
45. Yudi MB, Ajani AE, Andrianopoulos N, et al. Early versus delayed percutaneous coronary intervention in patients with non-ST elevation acute coronary syndromes. Coron Artery Dis. 2016;27(5):344-349. doi:10.1097/MCA.0000000000000374
46. Yavuz B, Ayturk M, Ozkan S, et al. A real world data of dabigatran etexilate: multicenter registry of oral anticoagulants in nonvalvular atrial fibrillation. J Thromb Thrombolysis. 2016;42(3):399-404. doi:10.1007/s11239-016-1361-4
47. Gitt AK, Bramlage P, Potthoff SA, et al. Azilsartan compared to ACE inhibitors in anti-hypertensive therapy: one-year outcomes of the observational EARLY registry. BMC Cardiovasc Disord. 2016;16:56. Published 2016 Mar 8. doi:10.1186/s12872-016-0222-6
48. Santos SM, Cunha S, Baptista R, et al. Early, real-world experience with direct oral anticoagulants in the treatment of intermediate-high risk acute pulmonary embolism. Rev Port Cardiol. 2017;36(11):801-806. doi:10.1016/j.repc.2017.01.010
49. Jungo P, Maul JT, Djamei V, et al. Superiority in Quality of Life Improvement of Biologics over Conventional Systemic Drugs in a Swiss Real-Life Psoriasis Registry. Dermatology. 2016;232(6):655-663. doi:10.1159/000455042
50. Blackburn R, Osborn D, Walters K, Falcaro M, Nazareth I, Petersen I. Statin prescribing for people with severe mental illnesses: a staggered cohort study of 'real-world' impacts. BMJ Open. 2017;7(3):e013154. Published 2017 Mar 7. doi:10.1136/bmjopen-2016-013154
51. Iketani R, Ide K, Yamada H, Kawasaki Y, Masaki N. The Safety Profile of Telaprevir-Based Triple Therapy in Clinical Practice: A Retrospective Cohort Study. Biol Pharm Bull. 2017;40(5):687-692. doi:10.1248/bpb.b16-00989
52. Jatene T, Castro-Filho A, Meneguz-Moreno RA, et al. Prospective comparison between three TAVR devices: ACURATE neo vs. CoreValve vs. SAPIEN XT. A single heart team experience in patients with severe aortic stenosis. Catheter Cardiovasc Interv. 2017;90(1):139-146. doi:10.1002/ccd.26837
53. Kang SH, Ahn JM, Lee CH, et al. Differential Event Rates and Independent Predictors of Long-Term Major Cardiovascular Events and Death in 5795 Patients With Unprotected Left Main Coronary Artery Disease Treated With Stents, Bypass Surgery, or Medication: Insights From a Large International Multicenter Registry. Circ Cardiovasc Interv. 2017;10(7):e004988. doi:10.1161/CIRCINTERVENTIONS.116.004988
54. Kosiborod M, Cavender MA, Fu AZ, et al. Lower Risk of Heart Failure and Death in Patients Initiated on Sodium-Glucose Cotransporter-2 Inhibitors Versus Other Glucose-Lowering Drugs: The CVD-REAL Study (Comparative Effectiveness of Cardiovascular Outcomes in New Users of Sodium-Glucose Cotransporter-2 Inhibitors). Circulation. 2017;136(3):249-259. doi:10.1161/CIRCULATIONAHA.117.029190
55. Sahlén A, Varenhorst C, Lagerqvist B, et al. Outcomes in patients treated with ticagrelor or clopidogrel after acute myocardial infarction: experiences from SWEDEHEART registry. Eur Heart J. 2016;37(44):3335-3342. doi:10.1093/eurheartj/ehw284
56. Almeida AM, Bedrosian C, Cole A, et al. Clinical benefit of eculizumab in patients with no transfusion history in the International Paroxysmal Nocturnal Haemoglobinuria Registry. Intern Med J. 2017;47(9):1026-1034. doi:10.1111/imj.13523
57. Piao ZH, Jin L, Kim JH, et al. Benefits of Statin Therapy in Patients With Acute Myocardial Infarction With Serum Low-Density Lipoprotein Cholesterol ≤ 50 mg/dl. Am J Cardiol. 2017;120(2):174-180. doi:10.1016/j.amjcard.2017.04.003
58. Crespo J, Calleja JL, Fernández I, et al. Real-World Effectiveness and Safety of Oral Combination Antiviral Therapy for Hepatitis C Virus Genotype 4 Infection. Clin Gastroenterol Hepatol. 2017;15(6):945-949.e1. doi:10.1016/j.cgh.2017.02.020
59. Li N, Betts KA, Messali AJ, Skup M, Garg V. Real-world Effectiveness of Biologic Disease-modifying Antirheumatic Drugs for the Treatment of Rheumatoid Arthritis After Etanercept Discontinuation in the United Kingdom, France, and Germany. Clin Ther. 2017;39(8):1618-1627. doi:10.1016/j.clinthera.2017.06.009
60. Weiss J, Force RW, Pugmire BA, et al. Comparative Effectiveness and Resource Usage in Patients Receiving First-line Taxane-based Chemotherapy for Stage IV Non-Small-cell Lung Cancer in a US Community Oncology Setting. Clin Lung Cancer. 2017;18(4):372-380.e1. doi:10.1016/j.cllc.2016.12.008
61. Lemmert ME, van Mieghem NM, van Geuns RJ, et al. The Promus Premier everolimus-eluting platinum chromium stent with durable polymer evaluated in a real world all-comer population in Rotterdam cardiology hospital (the P-SEARCH registry). Int J Cardiol. 2017;240:103-107. doi:10.1016/j.ijcard.2017.03.054
62. Calleja JL, Crespo J, Rincón D, et al. Effectiveness, safety and clinical outcomes of direct-acting antiviral therapy in HCV genotype 1 infection: Results from a Spanish real-world cohort. J Hepatol. 2017;66(6):1138-1148. doi:10.1016/j.jhep.2017.01.028
63. Almendro-Delia M, García-Alcántara Á, de la Torre-Prados MV, et al. Safety and Efficacy of Prasugrel and Ticagrelor in Acute Coronary Syndrome. Results of a "Real World" Multicenter Registry. Rev Esp Cardiol (Engl Ed). 2017;70(11):952-959. doi:10.1016/j.rec.2017.05.003
64. Fach A, Backhaus T, Schmucker J, et al. Bivalirudin versus heparin and provisional GP IIb/IIIa inhibitors in patients treated for ST-segment elevation myocardial infarctions: Comparison of outcomes in a "real-world" setting. J Interv Cardiol. 2017;30(4):301-308. doi:10.1111/joic.12397
65. Sung YK, Cho SK, Kim D, et al. Characteristics and outcomes of rheumatoid arthritis patients who started biosimilar infliximab. Rheumatol Int. 2017;37(6):1007-1014. doi:10.1007/s00296-017-3663-z
66. Iborra M, Pérez-Gisbert J, Bosca-Watts MM, et al. Effectiveness of adalimumab for the treatment of ulcerative colitis in clinical practice: comparison between anti-tumour necrosis factor-naïve and non-naïve patients [published correction appears in J Gastroenterol. 2017 Jul;52(7):875-877]. J Gastroenterol. 2017;52(7):788-799. doi:10.1007/s00535-016-1274-1
67. Rasia M, Solinas E, Marino M, et al. Comparison of 4 different strategies of DAPT after PCI in ACS real world population from a Northern Italy registry. J Thromb Thrombolysis. 2017;44(4):466-474. doi:10.1007/s11239-017-1567-0
68. Saumell Y, Sanchez L, González S, et al. Overall Survival of Patients with Locally Advanced or Metastatic Esophageal Squamous Cell Carcinoma Treated with Nimotuzumab in the Real World. Adv Ther. 2017;34(12):2638-2647. doi:10.1007/s12325-017-0631-7
69. Walker MS, Wong W, Ravelo A, Miller PJE, Schwartzberg LS. Effectiveness outcomes and health related quality of life impact of disease progression in patients with advanced nonsquamous NSCLC treated in real-world community oncology settings: results from a prospective medical record registry study. Health Qual Life Outcomes. 2017;15(1):160. Published 2017 Aug 14. doi:10.1186/s12955-017-0735-4
70. Gilligan AM, Waycaster CR, Bizier R, Chu BC, Carter MJ, Fife CE. Comparative Effectiveness of Clostridial Collagenase Ointment to Medicinal Honey for Treatment of Pressure Ulcers. Adv Wound Care (New Rochelle). 2017;6(4):125-134. doi:10.1089/wound.2016.0720
71. Kraus I, Sabolinski ML, Skornicki M, Parsons NB. The Comparative Effectiveness of a Human Fibroblast Dermal Substitute versus a Dehydrated Human Amnion/Chorion Membrane Allograft for the Treatment of Diabetic Foot Ulcers in a Real-world Setting. Wounds. 2017;29(5):125-132.
72. Mason KJ, Barker JNWN, Smith CH, et al. Comparison of Drug Discontinuation, Effectiveness, and Safety Between Clinical Trial Eligible and Ineligible Patients in BADBIR [published correction appears in JAMA Dermatol. 2018 Jul 1;154(7):852]. JAMA Dermatol. 2018;154(5):581-588. doi:10.1001/jamadermatol.2018.0183
73. Huang H, Bell KF, Gani R, Tugwell CW, Eudicone JM, Krukas-Hampel MR. A retrospective real-world study of dapagliflozin versus other oral antidiabetic drugs added to metformin in patients with type 2 diabetes. Am J Manag Care. 2018;24(8 Suppl):S132-S137.
74. Li X, Keshishian A, Hamilton M, et al. Apixaban 5 and 2.5 mg twice-daily versus warfarin for stroke prevention in nonvalvular atrial fibrillation patients: Comparative effectiveness and safety evaluated using a propensity-score-matched approach. PLoS One. 2018;13(1):e0191722. Published 2018 Jan 26. doi:10.1371/journal.pone.0191722
75. Chan AK, Bisson EF, Bydon M, et al. Laminectomy alone versus fusion for grade 1 lumbar spondylolisthesis in 426 patients from the prospective Quality Outcomes Database. J Neurosurg Spine. 2018;30(2):234-241. doi:10.3171/2018.8.SPINE17913
76. Schad F, Thronicke A, Steele ML, et al. Overall survival of stage IV non-small cell lung cancer patients treated with Viscum album L. in addition to chemotherapy, a real-world observational multicenter analysis [published correction appears in PLoS One. 2022 Aug 16;17(8):e0273387]. PLoS One. 2018;13(8):e0203058. Published 2018 Aug 27. doi:10.1371/journal.pone.0203058
77. Unni S, Wittbrodt E, Ma J, et al. Comparative effectiveness of once-weekly glucagon-like peptide-1 receptor agonists with regard to 6-month glycaemic control and weight outcomes in patients with type 2 diabetes. Diabetes Obes Metab. 2018;20(2):468-473. doi:10.1111/dom.13107
78. Jeon-Slaughter H, Khalili H, Tsai S, et al. Comparative Effectiveness Study of Drug-Eluting and Bare-Metal Peripheral Artery Stents in Endovascular Femoropopliteal Artery Revascularization. J Invasive Cardiol. 2018;30(10):373-379.
79. Tao YC, Deng R, Wang ML, et al. Satisfactory virological response and fibrosis improvement of sofosbuvir-based regimens for Chinese patients with hepatitis C virus genotype 3 infection: results of a real-world cohort study. Virol J. 2018;15(1):150. Published 2018 Oct 1. doi:10.1186/s12985-018-1066-8
80. Sabidó M, Hohenberger T, Grassi G. Pharmacological intervention in hypertension using beta-blockers: Real-world evidence for long-term effectiveness. Pharmacol Res. 2018;130:191-197. doi:10.1016/j.phrs.2018.01.010
81. Sangroongruangsri S, Chaikledkaew U, Kumluang S, et al. Real-World Safety of Intravitreal Bevacizumab and Ranibizumab Treatments for Retinal Diseases in Thailand: A Prospective Observational Study [published correction appears in Clin Drug Investig. 2019 Jan 19;:]. Clin Drug Investig. 2018;38(9):853-865. doi:10.1007/s40261-018-0678-5
82. Braune S, Grimm S, van Hövell P, et al. Comparative effectiveness of delayed-release dimethyl fumarate versus interferon, glatiramer acetate, teriflunomide, or fingolimod: results from the German NeuroTransData registry. J Neurol. 2018;265(12):2980-2992. doi:10.1007/s00415-018-9083-5
83. Kallenbach L, Shui AM, Cheng WY, et al. Predictors and Clinical Outcomes of Treatment Intensification in Patients With Type 2 Diabetes Uncontrolled on Basal Insulin in a Real-World Setting. Endocr Pract. 2018;24(9):805-814. doi:10.4158/EP-2017-0261
84. Almassi N, Gao T, Lee B, et al. Impact of Neoadjuvant Chemotherapy on Pathologic Response in Patients With Upper Tract Urothelial Carcinoma Undergoing Extirpative Surgery. Clin Genitourin Cancer. 2018;16(6):e1237-e1242. doi:10.1016/j.clgc.2018.08.003
85. Hiki N, Honda M, Etoh T, et al. Higher incidence of pancreatic fistula in laparoscopic gastrectomy. Real-world evidence from a nationwide prospective cohort study. Gastric Cancer. 2018;21(1):162-170. doi:10.1007/s10120-017-0764-z
86. Lang K, Nguyen H, Huang H, Bauer E, Levin P. Real-world clinical responses in patients with type 2 diabetes mellitus adding exenatide BID (EBID) or mealtime insulin to basal insulin: a retrospective study using electronic medical record data. Curr Med Res Opin. 2018;34(6):1045-1051. doi:10.1080/03007995.2018.1437027
87. Atsumi T, Fujio K, Yamaoka K, Tomobe M, Kuroyanagi K, Kameda H. Safety and effectiveness of subcutaneous tocilizumab in patients with rheumatoid arthritis in a real-world clinical setting. Mod Rheumatol. 2018;28(5):780-788. doi:10.1080/14397595.2017.1416760
88. Harrold LR, Litman HJ, Saunders KC, et al. One-year risk of serious infection in patients treated with certolizumab pegol as compared with other TNF inhibitors in a real-world setting: data from a national U.S. rheumatoid arthritis registry. Arthritis Res Ther. 2018;20(1):2. Published 2018 Jan 2. doi:10.1186/s13075-017-1496-5
89. López-Mínguez JR, Nogales-Asensio JM, Romani S, et al. TIOMAX: A Spanish Multicenter Registry of the real-world use of the TItanium OptiMAX® biostent: TIOMAX: Registro Español Multicéntrico Del Biostent De Titanio OptiMAX® En La Vida Real. Catheter Cardiovasc Interv. 2018;92(2):261-268. doi:10.1002/ccd.27326
90. Lichtenstein GR, Feagan BG, Cohen RD, et al. Infliximab for Crohn's Disease: More Than 13 Years of Real-world Experience. Inflamm Bowel Dis. 2018;24(3):490-501. doi:10.1093/ibd/izx072
91. Lee PH, Kwon O, Ahn JM, et al. Safety and Effectiveness of Second-Generation Drug-Eluting Stents in Patients With Left Main Coronary Artery Disease. J Am Coll Cardiol. 2018;71(8):832-841. doi:10.1016/j.jacc.2017.12.032
92. Huang HH, Wen YC, Chen HM, Hsiao FY, Ko BS. Rituximab maintenance improves overall survival in follicular lymphoma: A retrospective nationwide real-world analysis from Taiwan Cancer Registry Database. Cancer Med. 2018;7(8):3582-3591. doi:10.1002/cam4.1622
93. Zhao YJ, Teng M, Khoo AL, et al. A propensity score-matched comparison of biodegradable polymer vs second-generation durable polymer drug-eluting stents in a real-world population. Cardiovasc Ther. 2018;36(2):10.1111/1755-5922.12319. doi:10.1111/1755-5922.12319
94. Loughlin AM, Qiao Q, Nunes AP, et al. Effectiveness and Tolerability of Therapy With Once-Weekly Exenatide Versus Basal Insulin Among Injectable-Naive Patients With Type 2 Diabetes in a Real-World Setting in the United States. Diabetes Spectr. 2018;31(2):129-137. doi:10.2337/ds16-0081
95. Treadwell T, Sabolinski ML, Skornicki M, Parsons NB. Comparative Effectiveness of a Bioengineered Living Cellular Construct and Cryopreserved Cadaveric Skin Allograft for the Treatment of Venous Leg Ulcers in a Real-World Setting. Adv Wound Care (New Rochelle). 2018;7(3):69-76. doi:10.1089/wound.2017.0738
96. Fralick M, Kesselheim AS, Avorn J, Schneeweiss S. Use of Health Care Databases to Support Supplemental Indications of Approved Medications. JAMA Intern Med. 2018;178(1):55-63. doi:10.1001/jamainternmed.2017.3919
97. Hicks CW, Nejim B, Obeid T, Locham SS, Malas MB. Use of a primary carotid stenting technique does not affect perioperative outcomes. J Vasc Surg. 2018;67(6):1736-1743.e1. doi:10.1016/j.jvs.2017.09.056
98. Shibuya K, Ali KF, Ji X, et al. THE BENEFIT OF SHORT-TERM WEIGHT LOSS WITH ANTI-OBESITY MEDICATIONS IN REAL-WORLD CLINICAL PRACTICE. Endocr Pract. 2019;25(10):1022-1028. doi:10.4158/EP-2019-0081
99. Franchi M, Barni S, Tagliabue G, et al. Effectiveness of First-Line Bevacizumab in Metastatic Colorectal Cancer: The Observational Cohort Study GRETA. Oncologist. 2019;24(3):358-365. doi:10.1634/theoncologist.2017-0314
100. Kotani D, Kuboki Y, Horasawa S, et al. Retrospective cohort study of trifluridine/tipiracil (TAS-102) plus bevacizumab versus trifluridine/tipiracil monotherapy for metastatic colorectal cancer. BMC Cancer. 2019;19(1):1253. Published 2019 Dec 27. doi:10.1186/s12885-019-6475-6
101. Miura M, Yoshimura S, Sakai N, et al. Endovascular therapy for middle cerebral artery M2 segment occlusion: subanalyses of RESCUE-Japan Registry 2. J Neurointerv Surg. 2019;11(10):964-969. doi:10.1136/neurintsurg-2018-014627
102. Maguire FB, Morris CR, Parikh-Patel A, et al. First-Line Systemic Treatments for Stage IV Non-Small Cell Lung Cancer in California: Patterns of Care and Outcomes in a Real-World Setting. JNCI Cancer Spectr. 2019;3(3):pkz020. Published 2019 Apr 4. doi:10.1093/jncics/pkz020
103. Bottinor WJ, Shuey MM, Manouchehri A, et al. Renin-Angiotensin-Aldosterone System Modulates Blood Pressure Response During Vascular Endothelial Growth Factor Receptor Inhibition. JACC CardioOncol. 2019;1(1):14-23. doi:10.1016/j.jaccao.2019.07.002
104. Chang KC, Lee KY, Lu TH, et al. Opioid agonist treatment reduces losses in quality of life and quality-adjusted life expectancy in heroin users: Evidence from real world data. Drug Alcohol Depend. 2019;201:197-204. doi:10.1016/j.drugalcdep.2019.05.003
105. De Filippo O, Cortese M, D Ascenzo F, et al. Real-World Data of Prasugrel vs. Ticagrelor in Acute Myocardial Infarction: Results from the RENAMI Registry [published correction appears in Am J Cardiovasc Drugs. 2019 May 20;:]. Am J Cardiovasc Drugs. 2019;19(4):381-391. doi:10.1007/s40256-019-00339-3
106. Kuno T, Numasawa Y, Sawano M, et al. Real-world use of intravascular ultrasound in Japan: a report from contemporary multicenter PCI registry. Heart Vessels. 2019;34(11):1728-1739. doi:10.1007/s00380-019-01427-9
107. Bagley SJ, Talento S, Mitra N, et al. Comparative Effectiveness of Carboplatin/Pemetrexed With Versus Without Bevacizumab for Advanced Nonsquamous Non-Small Cell Lung Cancer. J Natl Compr Canc Netw. 2019;17(5):469-477. doi:10.6004/jnccn.2018.7102
108. Coleman CI, Pandya S, Wang L, et al. Treatment patterns, glycemic control and bodyweight with canagliflozin 300 mg versus GLP1RAs in Type II diabetes patients. J Comp Eff Res. 2019;8(11):889-905. doi:10.2217/cer-2019-0002
109. Buron MD, Chalmer TA, Sellebjerg F, et al. Comparative effectiveness of teriflunomide and dimethyl fumarate: A nationwide cohort study. Neurology. 2019;92(16):e1811-e1820. doi:10.1212/WNL.0000000000007314
110. Kumluang S, Ingsrisawang L, Sangroongruangsri S, et al. A real-world study of effectiveness of intravitreal bevacizumab and ranibizumab injection for treating retinal diseases in Thailand. BMC Ophthalmol. 2019;19(1):82. Published 2019 Mar 29. doi:10.1186/s12886-019-1086-1
111. Knauf W, Abenhardt W, Mohm J, et al. Similar effectiveness of R-CHOP-14 and -21 in diffuse large B-cell lymphoma-data from the prospective German Tumour Registry Lymphatic Neoplasms. Eur J Haematol. 2019;103(5):460-471. doi:10.1111/ejh.13295
112. Zhou FL, Watada H, Tajima Y, et al. Identification of subgroups of patients with type 2 diabetes with differences in renal function preservation, comparing patients receiving sodium-glucose co-transporter-2 inhibitors with those receiving dipeptidyl peptidase-4 inhibitors, using a supervised machine-learning algorithm (PROFILE study): A retrospective analysis of a Japanese commercial medical database. Diabetes Obes Metab. 2019;21(8):1925-1934. doi:10.1111/dom.13753
113. Olsen IC, Lie E, Vasilescu R, Wallenstein G, Strengholt S, Kvien TK. Assessments of the unmet need in the management of patients with rheumatoid arthritis: analyses from the NOR-DMARD registry. Rheumatology (Oxford). 2019;58(3):481-491. doi:10.1093/rheumatology/key338
114. Panés J, Lindsay JO, Teich N, et al. Five-year Safety Data From OPUS, a European Observational Safety Registry for Adults With Ulcerative Colitis Treated With Originator Infliximab [Remicade®] or Conventional Therapy. J Crohns Colitis. 2019;13(9):1148-1157. doi:10.1093/ecco-jcc/jjz048
115. Hee L, Gibbs OJ, Assad JG, et al. Real-world use of ticagrelor versus clopidogrel in percutaneous coronary intervention-treated ST-elevation myocardial infarction patients: A single-center registry study. J Saudi Heart Assoc. 2019;31(4):151-160. doi:10.1016/j.jsha.2019.05.005
116. Dumonteil N, Terkelsen C, Frerker C, et al. Outcomes of transcatheter aortic valve replacement without predilation of the aortic valve: Insights from 1544 patients included in the SOURCE 3 registry. Int J Cardiol. 2019;296:32-37. doi:10.1016/j.ijcard.2019.06.013
117. Choe JC, Cha KS, Ahn J, et al. Comparison of prescription rates and clinical outcomes in acute coronary syndrome patients who underwent percutaneous coronary intervention using different P2Y12 inhibitors in a large observational study. Int J Cardiol. 2019;274:21-26. doi:10.1016/j.ijcard.2018.09.011
118. Feld E, Harton J, Meropol NJ, et al. Effectiveness of First-line Immune Checkpoint Blockade Versus Carboplatin-based Chemotherapy for Metastatic Urothelial Cancer. Eur Urol. 2019;76(4):524-532. doi:10.1016/j.eururo.2019.07.032
119. Codreanu C, Popescu CC, Mogoșan C, et al. Efficacy and safety of original and biosimilar etanercept (SB4) in active rheumatoid arthritis - A comparison in a real-world national cohort. Biologicals. 2019;62:27-32. doi:10.1016/j.biologicals.2019.10.009
120. Casamira N, García-Camarero T, Montoro-Ronsano JB, et al. Use and Outcomes of Triple Antithrombotic Therapy with Non-Vitamin K Antagonists in Patients with Atrial Fibrillation Undergoing Percutaneous Coronary Intervention. Am J Med Sci. 2019;358(2):95-103. doi:10.1016/j.amjms.2019.03.009
121. Sotomi Y, Hirata A, Amiya R, et al. Bleeding Risk of Add-On Anti-Platelet Agents to Direct Oral Anticoagulants in Patients With Nonvalvular Atrial Fibrillation (From 2216 Patients in the DIRECT Registry). Am J Cardiol. 2019;123(8):1293-1300. doi:10.1016/j.amjcard.2019.01.027
122. Al-Gobari M, Agrinier N, Soudant M, Burnand B, Thilly N. Effects of Statins to Reduce All-Cause Mortality in Heart Failure Patients: Findings from the EPICAL2 Cohort Study. Am J Cardiovasc Drugs. 2019;19(5):497-508. doi:10.1007/s40256-019-00346-4
123. Takahashi N, Kojima T, Kida D, et al. Concomitant methotrexate has little effect on clinical outcomes of abatacept in rheumatoid arthritis: a propensity score matching analysis. Clin Rheumatol. 2019;38(9):2451-2459. doi:10.1007/s10067-019-04581-7
124. Madsen LR, Baggesen LM, Richelsen B, Thomsen RW. Effect of Roux-en-Y gastric bypass surgery on diabetes remission and complications in individuals with type 2 diabetes: a Danish population-based matched cohort study. Diabetologia. 2019;62(4):611-620. doi:10.1007/s00125-019-4816-2
125. Wanat MA, Wang X, Paranjpe R, et al. Warfarin vs. apixaban in nonvalvular atrial fibrillation, and analysis by concomitant antiarrhythmic medication use: A national retrospective study. Res Pract Thromb Haemost. 2019;3(4):674-683. Published 2019 Jun 17. doi:10.1002/rth2.12221
126. Rozemeijer R, van Muiden IG, Koudstaal S, et al. One-year clinical outcomes of patients treated with polymer-free amphilimus-eluting stents or zotarolimus-eluting stents: A propensity-score adjusted analysis. Catheter Cardiovasc Interv. 2019;94(1):61-69. doi:10.1002/ccd.28041
127. Desperak P, Hawranek M, Chodór PA, et al. Comparison of the everolimus-eluting bioresorbable vascular scaffold versus the everolimus-eluting metallic stent in real-world patients with ST-segment elevation myocardial infarction. Postepy Kardiol Interwencyjnej. 2020;16(1):49-57. doi:10.5114/aic.2020.93912
128. Rodríguez AJ, Ernst MT, Nybo M, et al. Oral Bisphosphonate use Reduces Cardiovascular Events in a Cohort of Danish Patients Referred for Bone Mineral Density. J Clin Endocrinol Metab. 2020;105(10):dgaa481. doi:10.1210/clinem/dgaa481
129. Man S, Ji X, Wang Y, et al. Add-On Effects of Conventional Synthetic Disease-Modifying Anti-Rheumatic Drugs in Ankylosing Spondylitis: Data from a Real-World Registered Study in China. Med Sci Monit. 2020;26:e921055. Published 2020 Jan 21. doi:10.12659/MSM.921055
130. Tan J, Yu W, Wu G, et al. A Real-World Study Comparing Various Antimicrobial Regimens for Bloodstream Infections Caused by Carbapenem-Resistant Gram-Negative Bacilli in a Tertiary Hospital, Shanghai, China, from 2010 to 2017. Infect Drug Resist. 2020;13:2453-2463. Published 2020 Jul 21. doi:10.2147/IDR.S247378
131. Qingwei Z, Dongsheng H, Duo L, et al. Fluorouracil Supplemented With Oxaliplatin or Irinotecan for Solid Tumors: Indications From Clinical Characteristics and Health Outcomes of Patients. Front Oncol. 2020;10:1542. Published 2020 Sep 2. doi:10.3389/fonc.2020.01542
132. Zubaid M, Khraishah H, Alahmad B, et al. Efficacy and Safety of Pharmacoinvasive Strategy Compared to Primary Percutaneous Coronary Intervention in the Management of ST-Segment Elevation Myocardial Infarction: A Prospective Country-Wide Registry. Ann Glob Health. 2020;86(1):13. Published 2020 Feb 5. doi:10.5334/aogh.2632
133. Volkova N, Moy K, Evans J, et al. Disease progression in patients with cystic fibrosis treated with ivacaftor: Data from national US and UK registries. J Cyst Fibros. 2020;19(1):68-79. doi:10.1016/j.jcf.2019.05.015
134. Jansson M, Själander S, Sjögren V, Renlund H, Norrving B, Själander A. Direct comparisons of effectiveness and safety of treatment with Apixaban, Dabigatran and Rivaroxaban in atrial fibrillation. Thromb Res. 2020;185:135-141. doi:10.1016/j.thromres.2019.11.010
135. Bounameaux H, Haas S, Farjat AE, et al. Comparative effectiveness of oral anticoagulants in venous thromboembolism: GARFIELD-VTE [published correction appears in Thromb Res. 2022 Aug;216:129]. Thromb Res. 2020;191:103-112. doi:10.1016/j.thromres.2020.04.036
136. Kleinecke C, Yu J, Neef P, et al. Clinical outcomes of Watchman vs. Amplatzer occluders for left atrial appendage closure (WATCH at LAAC). Europace. 2020;22(6):916-923. doi:10.1093/europace/euaa001
137. Svedbom A, Ståhle M. Real-world comparative effectiveness of adalimumab, etanercept and methotrexate: a Swedish register analysis. J Eur Acad Dermatol Venereol. 2020;34(3):525-532. doi:10.1111/jdv.15978
138. Lee S, Lee S, Jang IJ, Yu KS, Rhee SJ. Electronic medical records-based comparison of glycemic control efficacy between sulfonylureas and dipeptidyl peptidase-4 inhibitors added on to metformin monotherapy in patients with type 2 diabetes. Transl Clin Pharmacol. 2020;28(4):199-207. doi:10.12793/tcp.2020.28.e21
139. Park SJ, Kim J, Kim HS, et al. Real world effectiveness and safety of pegylated liposomal doxorubicin in platinum-sensitive recurrent ovarian, fallopian, or primary peritoneal cancer: a Korean multicenter retrospective cohort study. J Gynecol Oncol. 2020;31(2):e15. doi:10.3802/jgo.2020.31.e15
140. Ali MS, Ernst M, Robinson DE, et al. Alendronate use and bone mineral density gains in women with moderate-severe (stages 3B-5) chronic kidney disease: an open cohort multivariable and propensity score analysis from Funen, Denmark [published correction appears in Arch Osteoporos. 2021 Sep 17;16(1):135]. Arch Osteoporos. 2020;15(1):81. Published 2020 Jun 1. doi:10.1007/s11657-020-00746-z
141. Polavarapu NK, Kale R, Sethi B, et al. Effect of Gliclazide or Gliclazide plus Metformin Combination on Glycemic Control in Patients with T2DM in India: A Real-World, Retrospective, Longitudinal, Observational Study from Electronic Medical Records. Drugs Real World Outcomes. 2020;7(4):271-279. doi:10.1007/s40801-020-00206-7
142. Rubin KH, Möller S, Choudhury A, et al. Cardiovascular and skeletal safety of zoledronic acid in osteoporosis observational, matched cohort study using Danish and Swedish health registries. Bone. 2020;134:115296. doi:10.1016/j.bone.2020.115296
143. Stinchcombe TE, Miksad RA, Gossai A, Griffith SD, Torres AZ. Real-World Outcomes for Advanced Non-Small Cell Lung Cancer Patients Treated With a PD-L1 Inhibitor Beyond Progression. Clin Lung Cancer. 2020;21(5):389-394.e3. doi:10.1016/j.cllc.2020.04.008
144. Zhou Y, Zhang X, A R, Chen Y, Sun X. Could "triple-therapy" considered as a novel-optimal treatment model for acute bipolar depression? A prospective real-world research in China. J Psychiatr Res. 2020;131:220-227. doi:10.1016/j.jpsychires.2020.09.017
145. Pouwels XGLV, Geurts SME, Ramaekers BLT, et al. The relative effectiveness of eribulin for advanced breast cancer treatment: a study of the southeast Netherlands advanced breast cancer registry. Acta Oncol. 2020;59(1):82-89. doi:10.1080/0284186X.2019.1670356
146. Moser JC, Wei G, Colonna SV, Grossmann KF, Patel S, Hyngstrom JR. Comparative-effectiveness of pembrolizumab vs. nivolumab for patients with metastatic melanoma. Acta Oncol. 2020;59(4):434-437. doi:10.1080/0284186X.2020.1712473
147. Chari A, Richardson PG, Romanus D, et al. Real-world outcomes and factors impacting treatment choice in relapsed and/or refractory multiple myeloma (RRMM): a comparison of VRd, KRd, and IRd. Expert Rev Hematol. 2020;13(4):421-433. doi:10.1080/17474086.2020.1729734
148. Racey CS, Albert A, Donken R, et al. Cervical Intraepithelial Neoplasia Rates in British Columbia Women: A Population-Level Data Linkage Evaluation of the School-Based HPV Immunization Program. J Infect Dis. 2020;221(1):81-90. doi:10.1093/infdis/jiz422
149. Suwanai H, Watanabe R, Sato M, Odawara M, Matsumura H. Dipeptidyl Peptidase-4 Inhibitor Reduces the Risk of Developing Hypertrophic Scars and Keloids following Median Sternotomy in Diabetic Patients: A Nationwide Retrospective Cohort Study Using the National Database of Health Insurance Claims of Japan. Plast Reconstr Surg. 2020;146(1):83-89. doi:10.1097/PRS.0000000000006904
150. Liang JA, Tu CY, Hsia TC, Fang HY, Li CC, Chien CR. Effectiveness of image-guided radiotherapy for locally advanced lung cancer patients treated with definitive concurrent chemoradiotherapy. Thorac Cancer. 2020;11(9):2639-2649. doi:10.1111/1759-7714.13596
151. Shen Y, Zhou J, Shi L, et al. Effectiveness of sodium-glucose co-transporter-2 inhibitors on ischaemic heart disease. Diabetes Obes Metab. 2020;22(7):1197-1206. doi:10.1111/dom.14025
152. Longato E, Di Camillo B, Sparacino G, Gubian L, Avogaro A, Fadini GP. Cardiovascular outcomes of type 2 diabetic patients treated with SGLT-2 inhibitors versus GLP-1 receptor agonists in real-life. BMJ Open Diabetes Res Care. 2020;8(1):e001451. doi:10.1136/bmjdrc-2020-001451
153. Granqvist M, Burman J, Gunnarsson M, et al. Comparative effectiveness of dimethyl fumarate as the initial and secondary treatment for MS. Mult Scler. 2020;26(12):1532-1539. doi:10.1177/1352458519866600
154. Luo B, Kang H, Zhang H, et al. Pipeline Embolization device for intracranial aneurysms in a large Chinese cohort: factors related to aneurysm occlusion. Ther Adv Neurol Disord. 2020;13:1756286420967828. Published 2020 Nov 2. doi:10.1177/1756286420967828
155. Wang X, Zhang R, Du N, et al. An open label, multicenter, noninterventional study of apatinib in advanced gastric cancer patients (AHEAD-G202). Ther Adv Med Oncol. 2020;12:1758835920905424. Published 2020 Mar 19. doi:10.1177/1758835920905424
156. Matsuyama H, Matsubara N, Kazama H, Seto T, Tsukube S, Suzuki K. Real-world efficacy and safety of two doses of cabazitaxel (20 or 25 mg/m2) in patients with castration-resistant prostate cancer: results of a Japanese post-marketing surveillance study. BMC Cancer. 2020;20(1):649. Published 2020 Jul 13. doi:10.1186/s12885-020-07131-6
157. Zhang Y, Ding X, Hua B, et al. Real-world use of ACEI/ARB in diabetic hypertensive patients before the initial diagnosis of obstructive coronary artery disease: patient characteristics and long-term follow-up outcome. J Transl Med. 2020;18(1):150. Published 2020 Apr 1. doi:10.1186/s12967-020-02314-y
158. DeMichele A, Cristofanilli M, Brufsky A, et al. Comparative effectiveness of first-line palbociclib plus letrozole versus letrozole alone for HR+/HER2- metastatic breast cancer in US real-world clinical practice. Breast Cancer Res. 2021;23(1):37. Published 2021 Mar 24. doi:10.1186/s13058-021-01409-8
159. Dalle S, Mortier L, Corrie P, et al. Long-term real-world experience with ipilimumab and non-ipilimumab therapies in advanced melanoma: the IMAGE study. BMC Cancer. 2021;21(1):642. Published 2021 May 29. doi:10.1186/s12885-021-08032-y
160. Nabi H, Georgiadis S, Loft AG, et al. Comparative effectiveness of two adalimumab biosimilars in 1318 real-world patients with inflammatory rheumatic disease mandated to switch from originator adalimumab: nationwide observational study emulating a randomised clinical trial. Ann Rheum Dis. 2021;80(11):1400-1409. doi:10.1136/annrheumdis-2021-219951
161. Marsman D, Bolhuis T, Broeder ND, van den Hoogen F, den Broeder A, van der Maas A. Effect of add-on methotrexate in polymyalgia rheumatica patients flaring on glucocorticoids tapering: a retrospective study. Rheumatol Int. 2021;41(3):611-616. doi:10.1007/s00296-020-04783-2
162. Bianco M, Careggio A, Biolè CA, et al. Ticagrelor or Clopidogrel After an Acute Coronary Syndrome in the Elderly: A Propensity Score Matching Analysis from 16,653 Patients Treated with PCI Included in Two Large Multinational Registries. Cardiovasc Drugs Ther. 2021;35(6):1171-1182. doi:10.1007/s10557-021-07213-y
163. Dechering DG, Gonska BD, Brachmann J, et al. Efficacy and complications of cavo-tricuspid isthmus-dependent atrial flutter ablation in patients with and without structural heart disease: results from the German Ablation Registry. J Interv Card Electrophysiol. 2021;61(1):55-62. doi:10.1007/s10840-020-00769-z
164. Cohen SB, Greenberg JD, Harnett J, et al. Real-World Evidence to Contextualize Clinical Trial Results and Inform Regulatory Decisions: Tofacitinib Modified-Release Once-Daily vs Immediate-Release Twice-Daily for Rheumatoid Arthritis. Adv Ther. 2021;38(1):226-248. doi:10.1007/s12325-020-01501-z
165. Weissler EH, Annapureddy A, Wang Y, et al. Paclitaxel-coated devices in the treatment of femoropopliteal stenosis among patients ≥65 years old: An ACC PVI Registry Analysis. Am Heart J. 2021;233:59-67. doi:10.1016/j.ahj.2020.12.004
166. Yamamoto K, Shiomi H, Morimoto T, et al. Percutaneous Coronary Intervention Versus Coronary Artery Bypass Graftinge Among Patients with Unprotected Left Main Coronary Artery Disease in the New-Generation Drug-Eluting Stents Era (From the CREDO-Kyoto PCI/CABG Registry Cohort-3). Am J Cardiol. 2021;145:47-57. doi:10.1016/j.amjcard.2020.12.078
167. Zanghì A, Avolio C, Amato MP, et al. First-line therapies in late-onset multiple sclerosis: An Italian registry study. Eur J Neurol. 2021;28(12):4117-4123. doi:10.1111/ene.15006
168. Nannini M, Rizzo A, Nigro MC, et al. Standard versus personalized schedule of regorafenib in metastatic gastrointestinal stromal tumors: a retrospective, multicenter, real-world study. ESMO Open. 2021;6(4):100222. doi:10.1016/j.esmoop.2021.100222
169. Ueki K, Tanizawa Y, Nakamura J, et al. Long-term safety and efficacy of alogliptin, a DPP-4 inhibitor, in patients with type 2 diabetes: a 3-year prospective, controlled, observational study (J-BRAND Registry). BMJ Open Diabetes Res Care. 2021;9(1):e001787. doi:10.1136/bmjdrc-2020-001787
170. Mata J, Sanchís P, Valentí P, Hernández B, Aguilar JL. Treatment of headache disorders with acupuncture: a 6-year retrospective study. Acupunct Med. 2021;39(5):452-460. doi:10.1177/0964528420987571
171. Thaler FS, Zimmermann L, Kammermeier S, et al. Rituximab Treatment and Long-term Outcome of Patients With Autoimmune Encephalitis: Real-world Evidence From the GENERATE Registry. Neurol Neuroimmunol Neuroinflamm. 2021;8(6):e1088. Published 2021 Oct 1. doi:10.1212/NXI.0000000000001088
172. Patel T, Miccio J, Cecchini M, et al. Clinical outcomes of first line FOLFIRINOX vs. gemcitabine plus nab-paclitaxel in metastatic pancreatic cancer at the Yale Smilow Hospital System. J Gastrointest Oncol. 2021;12(6):2547-2556. doi:10.21037/jgo-21-202
173. von Mehren M, Heinrich MC, Shi H, et al. Clinical efficacy comparison of avapritinib with other tyrosine kinase inhibitors in gastrointestinal stromal tumors with PDGFRA D842V mutation: a retrospective analysis of clinical trial and real-world data. BMC Cancer. 2021;21(1):291. Published 2021 Mar 19. doi:10.1186/s12885-021-08013-1
174. Kjaer SK, Dehlendorff C, Belmonte F, Baandrup L. Real-World Effectiveness of Human Papillomavirus Vaccination Against Cervical Cancer. J Natl Cancer Inst. 2021;113(10):1329-1335. doi:10.1093/jnci/djab080
175. Omland LH, Stormoen DR, Dohn LH, et al. Real-World Study of Treatment with Pembrolizumab Among Patients with Advanced Urothelial Tract Cancer in Denmark. Bladder Cancer. 2021;7(4):413-425. doi:10.3233/BLC-211523
176. Chung CH, Bonomi M, Steuer CE, et al. Concurrent Cetuximab and Nivolumab as a Second-Line or beyond Treatment of Patients with Recurrent and/or Metastatic Head and Neck Squamous Cell Carcinoma: Results of Phase I/II Study. Cancers (Basel). 2021;13(5):1180. Published 2021 Mar 9. doi:10.3390/cancers13051180
177. Shouval A, Lidar M, Reitblat T, et al. Real-world effectiveness of tofacitinib in patients with rheumatoid arthritis: a prospective observational study. Clin Exp Rheumatol. 2021;39(6):1378-1384. doi:10.55563/clinexprheumatol/do2uxu
178. Curtis JR, Palmer JL, Reed GW, et al. Real-World Outcomes Associated With Methotrexate, Sulfasalazine, and Hydroxychloroquine Triple Therapy Versus Tumor Necrosis Factor Inhibitor/Methotrexate Combination Therapy in Patients With Rheumatoid Arthritis. Arthritis Care Res (Hoboken). 2021;73(8):1114-1124. doi:10.1002/acr.24253
179. Lorscheider J, Benkert P, Lienert C, et al. Comparative analysis of dimethyl fumarate and fingolimod in relapsing-remitting multiple sclerosis. J Neurol. 2021;268(3):941-949. doi:10.1007/s00415-020-10226-6
180. Brufsky A, Liu X, Li B, McRoy L, Layman RM. Real-World Tumor Response of Palbociclib Plus Letrozole Versus Letrozole for Metastatic Breast Cancer in US Clinical Practice [published correction appears in Target Oncol. 2021 Nov;16(6):865]. Target Oncol. 2021;16(5):601-611. doi:10.1007/s11523-021-00826-1
181. Hoffman V, Hallas J, Linder M, et al. Cardiovascular Risk in Users of Mirabegron Compared with Users of Antimuscarinic Treatments for Overactive Bladder: Findings from a Non-Interventional, Multinational, Cohort Study. Drug Saf. 2021;44(8):899-915. doi:10.1007/s40264-021-01095-7
182. Andreano A, Bergamaschi W, Russo AG. Immune checkpoint inhibitors at any treatment line in advanced NSCLC: Real-world overall survival in a large Italian cohort. Lung Cancer. 2021;159:145-152. doi:10.1016/j.lungcan.2021.06.019
183. Richter S, Ebert M, Bertagnolli L, et al. Impact of electroanatomical mapping-guided lead implantation on procedural outcome of His bundle pacing. Europace. 2021;23(3):409-420. doi:10.1093/europace/euaa292
184. Karachiwala H, Tilley D, Abdel-Rahman O, Morris D. Comparison of oral versus intravenous etoposide in the management of small-cell lung cancer; A real-world, population-based study. Clin Respir J. 2021;15(1):36-41. doi:10.1111/crj.13266
185. Mori S, Urata Y, Yoshitama T, Ueki Y. Tofacitinib versus tocilizumab in the treatment of biological-naïve or previous biological-failure patients with methotrexate-refractory active rheumatoid arthritis. RMD Open. 2021;7(2):e001601. doi:10.1136/rmdopen-2021-001601
186. Guery B, Berger P, Gauzit R, et al. A prospective, observational study of fidaxomicin use for Clostridioides difficile infection in France. J Int Med Res. 2021;49(6):3000605211021278. doi:10.1177/03000605211021278
187. Charles Crozafon P, Bouchet C, Zignani M, et al. Comparison of real-world treatment outcomes of femtosecond laser-assisted cataract surgery and phacoemulsification cataract surgery: A retrospective, observational study from an outpatient clinic in France. Eur J Ophthalmol. 2021;31(4):1809-1816. doi:10.1177/1120672120925766
